# Bloom helicase mediates formation of large single–stranded DNA loops during DNA end processing

**DOI:** 10.1038/s41467-022-29937-7

**Published:** 2022-04-26

**Authors:** Chaoyou Xue, Sameer J. Salunkhe, Nozomi Tomimatsu, Ajinkya S. Kawale, Youngho Kwon, Sandeep Burma, Patrick Sung, Eric C. Greene

**Affiliations:** 1 Department of Biochemistry and Molecular Biophysics, Columbia University, New York, NY 10032 USA; 2 Department of Biochemistry and Structural Biology, University of Texas Health Science Center at San Antonio, San Antonio, TX 78229 USA; 3 The Greehey Children’s Cancer Research Institute, University of Texas Health Science Center at San Antonio, San Antonio, TX 78229 USA; 4 Tianjin Institute of Industrial Biotechnology, Chinese Academy of Sciences, Tianjin, 300308 China; 5 Department of Neurosurgery, University of Texas Health Science Center at San Antonio, San Antonio, TX 78229 USA; 6 Present Address: Tianjin Institute of Industrial Biotechnology, Chinese Academy of Sciences, Tianjin, 300308 China; 7 Present Address: Massachusetts General Hospital Cancer Center, Harvard Medical School, Charlestown, MA 02129 USA

**Keywords:** Single-molecule biophysics, Homologous recombination

## Abstract

Bloom syndrome (BS) is associated with a profoundly increased cancer risk and is caused by mutations in the Bloom helicase (BLM). BLM is involved in the nucleolytic processing of the ends of DNA double–strand breaks (DSBs), to yield long 3′ ssDNA tails that serve as the substrate for break repair by homologous recombination (HR). Here, we use single–molecule imaging to demonstrate that BLM mediates formation of large ssDNA loops during DNA end processing. A BLM mutant lacking the N–terminal domain (NTD) retains vigorous in vitro end processing activity but fails to generate ssDNA loops. This same mutant supports DSB end processing in cells, however, these cells do not form RAD51 DNA repair foci and the processed DSBs are channeled into synthesis–dependent strand annealing (SSA) instead of HR–mediated repair, consistent with a defect in RAD51 filament formation. Together, our results provide insights into BLM functions during homologous recombination.

## Abstract (web summary)

Bloom syndrome is a genetic disorder associated with increased cancer risk and is caused by mutations in Bloom helicase. This study investigates the mechanisms used by BLM helicase as initiates the repair of broken chromosomes.

## Introduction

Homologous recombination (HR) is an important pathway for repairing DNA double-strand breaks (DSBs), single-strand gaps, and stalled or collapsed replication forks^1–5^. Aberrant HR underlies the chromosomal rearrangements often associated with cancers, cancer prone syndromes, and numerous genetic diseases^1–5^. During the early stages of HR, DSB ends are processed by 5′→3′ strand resection, yielding 3′ single-stranded DNA (ssDNA) overhangs that become coated with the heterotrimeric ssDNA-binding protein replication protein A (RPA). RPA is then replaced by the ATP-dependent recombinase RAD51 to form a nucleoprotein filament, termed the presynaptic complex, capable of catalyzing DNA strand invasion during which the RAD51-bound ssDNA is paired with a homologous double-stranded DNA (dsDNA) donor template^4,5^. DNA synthesis then occurs within the resulting D-loop structure, and repair is completed via one of several mechanistically distinct pathways^1–5^.

In addition to creating a ssDNA template for RAD51 presynaptic complex assembly, extensive DNA end resection also plays a crucial role in committing break repair via HR rather than nonhomologous end joining^6–8^. In humans, long-range DNA end resection is catalyzed by the Bloom helicase (BLM; 1417 amino acids) in combination with either DNA2 or EXO1^6–10^. Germline mutations in BLM are associated with Bloom syndrome, an autosomal recessive genetic disorder characterized by severe developmental defects and strong cancer predisposition^11–13^. Moreover, BLM and RAD51 are often overexpressed in many cancer types^14^. In addition to its DNA end processing role, BLM has been implicated in the disruption of inactivated ADP-bound RAD51 filaments and strand invasion intermediates^15–17^, Topoisomerase IIIα–dependent dissolution of the double Holliday junction (dHJ) that arises during some HR events^18^, and in the restart of stalled or collapsed replication forks^19–23^. Given its importance to the maintenance of genome integrity, BLM has emerged as a potential target for anticancer chemotherapeutics^24,25^.

BLM (1417 amino acid residues) belongs to the highly conserved RecQ subgroup of the super-family 2 helicases^24,26–28^. BLM has DNA-dependent ATPase and 3′→5′ helicase activities and is capable of unwinding DNA structures mimicking a variety of DNA replication and repair intermediates^26,27,29^. In addition to its helicase core domain, BLM harbors a RecQ C-terminal (RQC) domain that confers high-affinity structure-specific DNA-binding activity, and a helicase- and RNaseD-like C-terminal (HRDC) domain that promotes BLM recruitment to DNA damage^30,31^. In vitro studies have provided insights into BLM activities relevant for its involvement in DNA end resection^6^ and dHJ dissolution^18^. However, our current understanding of BLM function(s) remains relatively limited at the mechanistic level. Given that BLM is indispensable for multiple aspects of genome integrity, it remains challenging to fully define how BLM mutations impact any one particular process.

Here, we used total internal reflection fluorescence microscopy (TIRFM) to define the interactions of N-terminal domain (NTD) and C-terminal domain (CTD) deletion BLM mutants with DNA. Our results confirm that the NTD is involved in BLM oligomerization and also show that the NTD allows BLM to generate a large ssDNA loop while unwinding duplex DNA. Surprisingly, while an NTD truncation mutant loses the ability to oligomerize, it is more proficient than full-length BLM at DNA end resection in vitro. However, cells expressing this NTD truncation mutant are sensitive to DNA damaging agents, and although these cells undergo DNA end processing, they are defective for DNA damage-induced RAD51 focus formation and the processed DSBs are channeled into the synthesis-dependent strand annealing (SSA) repair pathway instead of HR-mediated repair. We also demonstrate that there is a region within the CTD that is necessary for targeting BLM to DNA ends. Even though deleting amino acid residues of BLM needed for DNA end recognition has little or no impact on translocase and helicase activities, the CTD mutant fails to selectively bind DNA ends, which leads to a defect in end resection activity both in vitro and in vivo. Together, our study provides insights regarding the multi-faceted role of the BLM helicase in DSB repair by HR.

## Results

### DNA end resection by human BLM–DNA2–RPA

**Fig. 1:**
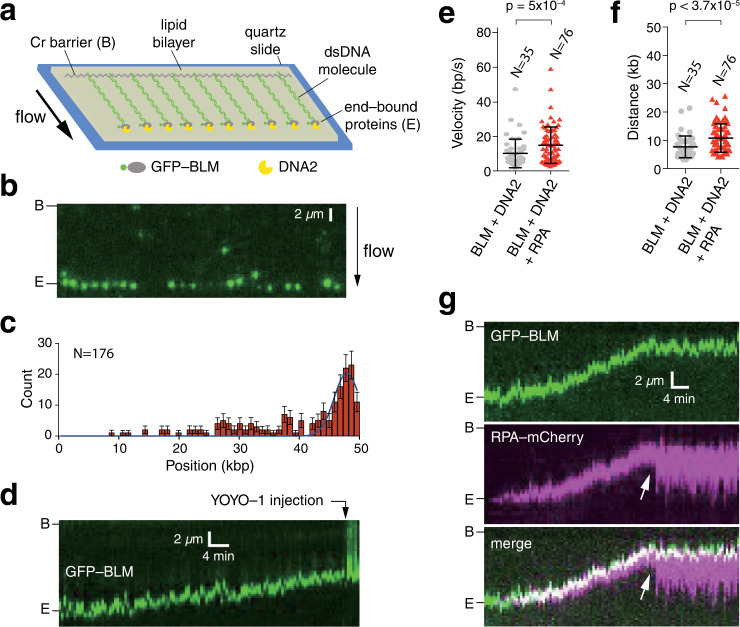
Generation of ssDNA during DNA end resection. **a** Schematic of BLM-mediated end resection assay using single-tethered dsDNA curtains. **b** Wide-field TIRFM image showing GFP-BLM (green) bound to the free DNA ends. **c** Binding distribution of GFP-BLM on the DNA (*N* = 176). The height of each bar represents the cumulative number of GFP-BLM molecules bound within each given bin and error bars represent 95% confidence intervals (CI) calculated from bootstrap analysis. **d** Kymograph showing the dsDNA end resection in reactions where end-bound GFP-BLM (0.2 nM) was chased with 0.2 nM DNA2. The dsDNA was stained with 0.5 nM YOYO-1 (green) at the time point indicated with an arrowhead. **e** Velocity distributions of end resection reactions with GFP-BLM and DNA2 plus or minus RPA. The center bar represents the mean of the data and error bars represent standard deviation (SD). Also see Supplementary Table 1. **f** End resection processivity in reactions with GFP-BLM and DNA2 plus or minus RPA. The center bar represents the mean of the data and error bars represent SD. Also see Supplementary Table 1. **g** Kymograph showing DNA end resection in assays with 0.2 nM GFP-BLM (green), 0.2 nM DNA2 (unlabeled) and 2 nM RPA-mCherry (magenta). The white arrowhead highlights spontaneous release of the 3′ ssDNA end.

We used single-molecule DNA curtain assays to visualize DNA end resection in reactions containing BLM, the helicase/nuclease DNA2 and the ssDNA-binding protein RPA. In these assays, we used double-stranded λ–DNA (48.5 kb) molecules with a 30-nucleotide (nt) ssDNA 3′ overhang as a substrate, which mimics a partially processed DSB based upon the preference of BLM for 3′ ssDNA overhangs during dsDNA unwinding^9,32–35^. DNA molecules were anchored via a biotin-streptavidin linkage to a lipid bilayer deposited onto a flow-cell surface and aligned at nanofabricated chromium (Cr) barriers using buffer flow, as previously described (Fig. 1a)^36^. BLM was expressed as a GFP fusion protein to allow us to visualize it in the DNA curtain assays by TIRFM. Previous studies have shown that GFP-BLM is functional both in vitro and in vivo^17,23,37,38^. When GFP-BLM was injected into the flowcell with 2 mM ATP, the majority of the GFP-BLM molecules (60%, *N* = 105 of 176) co-localized with the free end of DNA molecules (Fig. 1b, c). Photobleaching step experiments suggested that BLM bound to the DNA ends mainly as a heterogeneous multimer ranging from 1 to 6 monomers in size, with an average of 3.6 ± 1.5 (*N* = 109) BLM monomers bound per DNA end (Supplementary Fig. 1a, b). The end-bound BLM complexes were highly stable, exhibiting a half-life that exceeded the 20-min duration of the measurements (Supplementary Fig. 1c). We have previously shown that the helicase Sgs1, the budding yeast BLM ortholog, binds DNA ends as well, but it is promptly displaced from the 30-nt 3′ overhang by RPA^35^. In contrast, we found that human BLM is not removed from DNA ends when chased with RPA, highlighting a major difference in the end-binding behavior of BLM compared to Sgs1 (Supplementary Fig. 1c)^35^.

As we and others have previously reported, GFP-BLM readily undergoes translocation when bound to an internal position of a dsDNA molecule^17,39^. When DNA2 was injected into the flow cell, the end-bound GFP-BLM translocated away from DNA ends at rate of 10 ± 8 bp s^–1^ (*N* = 48) and traveled an average distance of 7.1 ± 2.7 kb (*N* = 48) before stopping (Fig. 1e, f and Supplementary Table 1). These values increased to 15 ± 10 bp s^–1^ (*N* = 76) and 10.8 ± 5.0 kb (*N* = 76) in reactions that included both DNA2 and RPA (Fig. 1e, f and Supplementary Table 1), corresponding to 50% increase in velocity and 52% increase in processivity. In the absence of RPA or DNA2, only 7.4% (*N* = 23/309) of observed end-bound BLM molecules underwent translocation within the observation time windows. However, 20.3% (*N* = 48/237) and 25.5% (*N* = 64/251) of observed BLM translocated on the DNA when either DNA2 or RPA were present, respectively. In reactions containing both DNA2 and RPA, 36.9% (*N* = 76/206) of the end-bound BLM underwent translocation. The translocation velocity and processivity of GFP-BLM, and the stimulatory effects of DNA2 and RPA on these parameters, were comparable to previous reported values^40^.

Assays in which the DNA was labeled with the intercalating fluorescent dye YOYO-1, either before or after addition of DNA2, revealed that the length of DNA molecules decreased in the presence of BLM and DNA2, confirming that the DNA was nucleolytically cleaved through the action of DNA2, as anticipated (Fig. 1d). We note that YOYO-1 did not drastically affect the BLM translocation velocity (8 ± 6 bp s^–1^ with YOYO-1 vs. 10 ± 8 bp s^–1^ without YOYO-1; *N* = 35; *p* = 0.20), however, the processivity of end resection was lower when YOYO-1 was present (5.7 ± 3.2 kb with YOYO-1 vs. 7.1 ± 2.7 kb without YOYO-1; *N* = 35; *p* = 0.017) (Supplementary Fig. 1d, e and Supplementary Table 1). Consistent with previous results^9,41–43^, we found no evidence for extensive DNA end resection in assays with DNA2 alone (Supplementary Fig. 1f) and end resection was also abolished in control assays using a helicase deficient BLM mutant (BLM–K695A; Supplementary Fig. 1g). Taken together, these findings suggest that GFP-BLM binds to the 30-nt 3′ ssDNA overhangs at the dsDNA ends and is able to support efficient end processing in the presence of DNA2 and RPA.

### RPA co-localization with the processed DNA ends

DSB processing is expected to yield a long 3′ ssDNA overhang which can be rapidly bound by RPA^2,4,5^. Therefore, we next included mCherry-labeled RPA in reactions with end-bound GFP-BLM and unlabeled DNA2 to directly visualize the ssDNA product of end resection. As anticipated, we could readily distinguish the binding of RPA-mCherry to the DNA as it was being processed by BLM–DNA2 (Fig. 1g), and the RPA-mCherry intensity signal increased substantially over time, indicating the continuous generation of ssDNA (Supplementary Fig. 2a).

Interestingly, the RPA-bound ssDNA overhangs were not elongated, but instead appeared as bright, highly condensed puncta that tracked closely with the moving GFP-BLM signal (Fig. 1g). The majority of the RPA-mCherry puncta remained highly condensed and co-localized with GFP-BLM for the entire 30-min duration of the experiments (72.2%, *N* = 44 of 61). However, some of the end-bound RPA-mCherry puncta underwent extension abruptly in a single-step event (27.8%, *N* = 17 of 61; Fig. 1g) and the final length of the released RPA–ssDNA complexes was positively correlated to the distance over which GFP-BLM had traveled on the dsDNA (Supplementary Fig. 2b). These single-step elongation events suggested that the ssDNA may have formed a condensed loop or loop-like structure that in some cases was spontaneously released during end resection.

### Formation of a 3′ ssDNA loop during DNA end resection

**Fig. 2:**
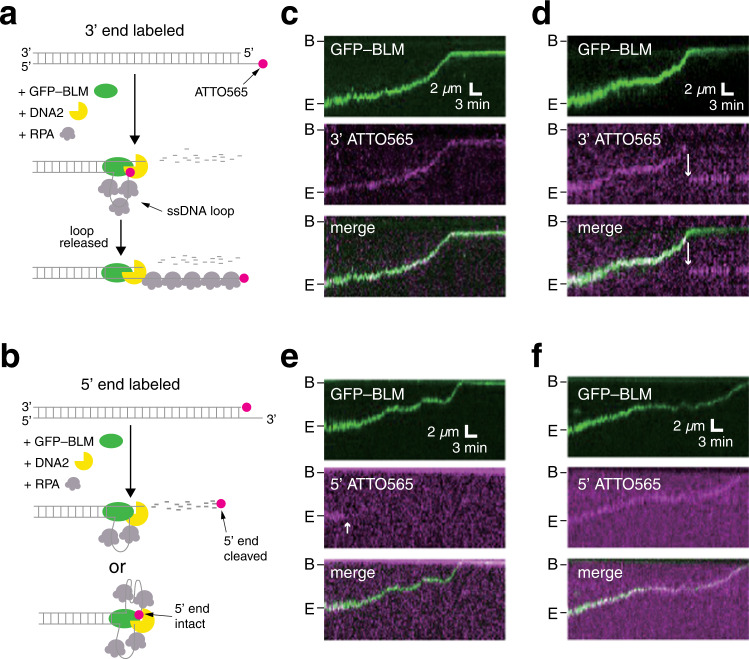
Fate of the 3′ and 5′ DNA ends during end resection. **a** Schematic of end resection with a 3′ ATTO565 end-labeled DNA substrate. **b** Schematic of end resection with a 5′ ATTO565 end-labeled DNA substrate. **c** Kymograph showing an example where the 3′ ATTO565 label (magenta) tracks with GFP-BLM (green) during DNA end resection. **d** Kymograph showing an example where the 3′ ATTO565 label (magenta) initially tracks with GFP-BLM (green) during DNA end resection but is then released from the protein complex; release of the end is highlighted with an arrowhead. **e** Kymograph showing an example where the 5′ ATTO565 label (magenta) is lost at the onset of DNA end resection. **f** Kymograph showing an example where the 5′ ATTO565 label (magenta) tracks with GFP-BLM (green) during end resection. All reactions contained 0.2 nM GFP-BLM, 0.2 nM DNA2 and 2 nM RPA.

Our data suggested that the 3′ end of the ssDNA overhang produced during DNA end resection might remain bound by the BLM–DNA2 complex as it moved along the dsDNA. To test this possibility, either the 3′ or the 5′ ends of the λ-DNA (48.5 kb) molecules were labeled with ATTO565, allowing us to track the fate of the DNA ends (Fig. 2a, b).

Most GFP-BLM molecules (71.3%, *N* = 102 of 143 for 3′-end; 80.0%, *N* = 107 of 134 for 5′-end) co-localized with the free DNA ends for both 3′ and 5′ ATTO565 end-labeled substrates, indicating that the dye does not affect end recognition by BLM (Supplementary Fig. 3a–d). For the 3′-end-labeled substrate, in reactions with GFP-BLM and unlabeled RPA and unlabeled DNA2, the ATTO565 dye co-localized with the GFP-BLM while it translocated along the DNA for all observed molecules (100%, *N* = 86; Fig. 2c). Most of these molecules showed no evidence for sudden release of the DNA ends (99%, *N* = 85 of 86; Fig. 2c). However, in one case, the ATTO565-labeled 3′ end became disengaged from GFP-BLM, consistent with the formation and sudden release of a ssDNA loop (Fig. 2d). Interestingly, there was a marked difference in the fraction of 3′ ssDNA loops that were spontaneously released in reactions with RPA-mCherry (27.8% released, Fig. 1g) compared to reactions with unlabeled RPA (1% released, Fig. 2d). These findings imply that the ssDNA loops are highly stable and very few undergo spontaneous release in reactions with wild-type RPA.

In striking contrast to the 3′ labeled DNA substrate, the majority of the 5′-end-labeled ATTO565 (87.5%, *N* = 105 of 120) quickly disappeared (Fig. 2e). The disappearance of the 5′ label was consistent with the expectation that it was cleaved off by the nuclease activity of DNA2 during the end processing reaction (Fig. 2e). In a smaller fraction of cases (12.5%, *N* = 15/120), the 5′ end-labeled ATTO565 traveled together with GFP-BLM, indicating that it was not cleaved by DNA2 (Fig. 2f); it is possible that this in this subset of cases DNA2 was absent from the end-bound complexes. Taken together, our results indicate that the 3′ ssDNA overhangs generated through 5′-strand resection remain in contact with the translocating BLM–DNA2 complex to yield a large loop of ssDNA.

### BLM helicase mediates formation of the ssDNA loop

**Fig. 3:**
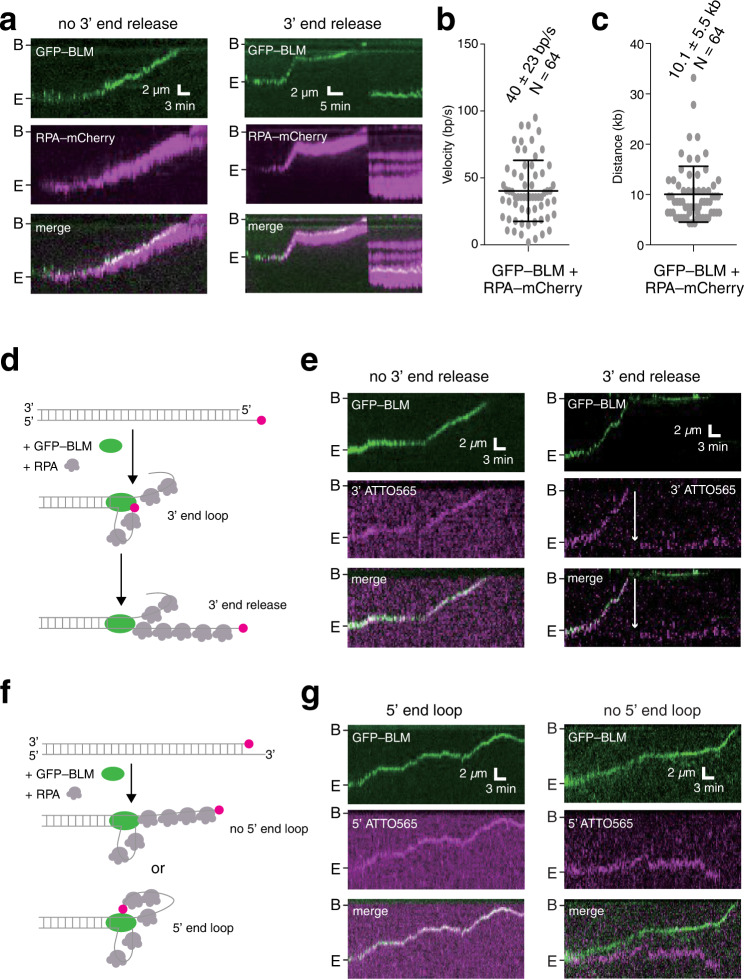
DNA2 is not necessary for ssDNA looping. **a** Kymographs showing examples with and without evidence for 3′ ssDNA loop release in reactions with 0.2 nM GFP-BLM (green) and 2 nM RPA-mCherry (magenta). **b** Velocity distribution for GFP-BLM in reactions with RPA-mCherry. The center bar represents the mean of the data and error bars represent SD. Also see Supplementary Table 1. **c** Processivity of GFP-BLM in reactions with RPA-mCherry. The center bar represents the mean of the data and error bars represent SD. Also see Supplementary Table 1. **d** Schematic of reaction with GFP-BLM and RPA with a 3′ ATTO565-labeled DNA substrate. **e** Kymographs showing examples where the 3′ ATTO565-labeled ssDNA end (magenta) is either released or not released in reactions with GFP-BLM (green) and unlabeled RPA. Note, 3′ end release is highlighted with a white arrowhead. **f** Schematic of reaction with GFP-BLM and RPA with a 5′ ATTO565-labeled DNA. **g** Kymographs showing examples where the 5′ ATTO565-labeled ssDNA end (magenta) either does or does not track with the GFP-BLM (green) in reactions with unlabeled RPA.

Next, we sought to determine if BLM alone was sufficient for ssDNA loop formation in the absence of DNA2. For these experiments, we injected RPA-mCherry into a sample chamber containing end-bound GFP-BLM in buffer with 2 mM ATP. As expected, the RPA-mCherry signal increased with time indicating that BLM translocation coincided with the continuous generation of ssDNA. Importantly, the RPA-mCherry signal remained in highly condensed puncta that co-localized with GFP-BLM (Fig. 3a), and analysis of the reaction trajectories yielded a translocation velocity of 40 ± 23 bp s^–1^ (*N* = 64) and processivity of 10.1 ± 5.5 kb (*N* = 64; Fig. 3b, c). The more rapid translocation of BLM in the absence of DNA2 was consistent with previous data demonstrating that DNA2 slowed the movement of BLM^40^. Among the GFP-BLM translocation events observed in the absence of DNA2, 57.5% of the RPA-mCherry puncta (*N* = 23/40) remained closely co-localized with GFP-BLM for the 30-min duration of our observations, with the remainder showing evidence of 3′ end release (Fig. 3a). Notably, the fraction of spontaneously released 3′ ssDNA loops was markedly higher in these reactions containing GFP-BLM and RPA-mCherry (42.5%, *N* = 17/40) compared to reactions that contained GFP-BLM, RPA-mCherry and DNA2 (27.8%, *N* = 17/61), suggesting that DNA2 may also contribute to continued retention of the 3′ ssDNA end during resection.

We further analyzed ssDNA looping events in reactions without DNA2 using 3′ or 5′ ATTO565 end-labeled substrates and unlabeled RPA. For the 3′ end-labeled substrate (Fig. 3d), ATTO565 co-localized with GFP-BLM as it translocated along the DNA in all the events (*N* = 69; Fig. 3e). In most cases (97.1%, *N* = 67/69), the 3′ end remained co-localized with GFP-BLM throughout the reaction (Fig. 3e). Rarely (2.9%, *N* = 2 of 69), the 3′ end was released suddenly from GFP-BLM (Fig. 3e). With the 5′ end-labeled substrate (Fig. 3f), the ATTO565 dye was not cleaved from the DNA when DNA2 was absent from the reactions, as expected, and instead the intact 5′ end co-localized with the translocating GFP-BLM in 52.6% of the observed events (*N* = 30/57; Fig. 3g). For the remaining events (47.4%, *N* = 27/57), the 5′ end of the DNA did not co-localize with GFP-BLM as reflected by a clear spatial separation between the ATTO565 and GFP signals (Fig. 3g). Together, these results indicate that upon DNA strand separation, BLM alone can engage the newly unwound 3′ ssDNA strand to allow for ssDNA loop formation.

### The BLM N-terminal domain regulates oligomerization

**Fig. 4:**
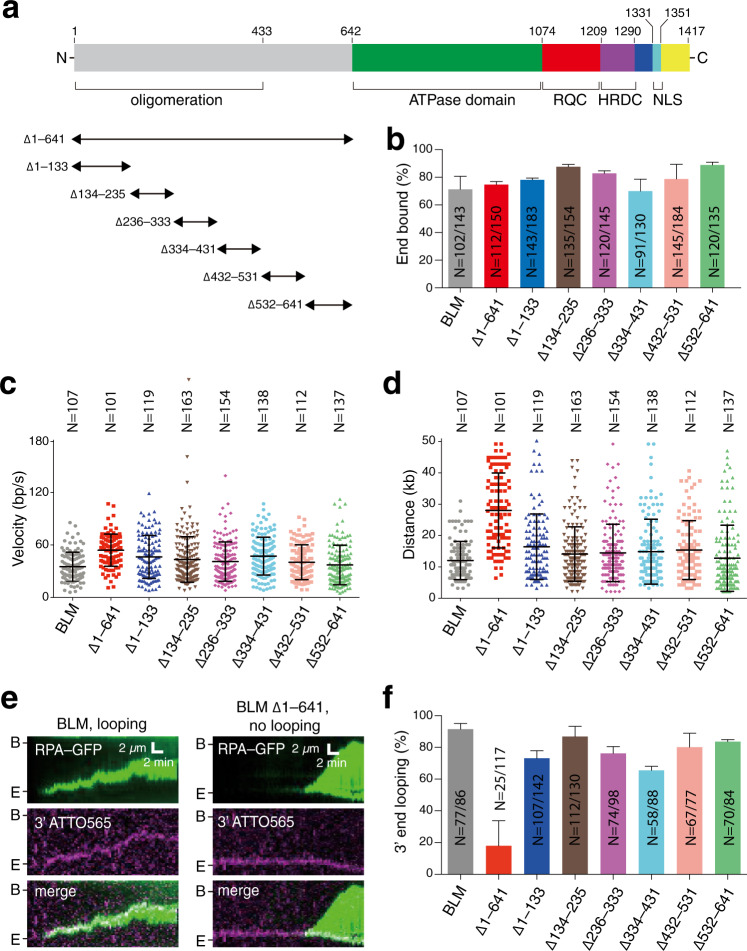
The N-terminal domain of BLM is required for ssDNA loop formation. **a** Schematic showing the domain organization of BLM. Black arrowheads indicate the NTD truncation mutants analyzed in this study. **b** Fraction of DNA end-bound complexes for each BLM mutant. The height of each bar represents the percentage end-bound BLM molecules and error bars represent 95% confidence intervals (CI) calculated from bootstrap analysis. Also see Supplementary Fig. 4. **c** Velocity distributions for the BLM NTD truncation mutants compared to full-length BLM. Reactions contained 0.2 nM BLM (or the indicated BLM mutant) and 2 nM RPA but did not contain DNA2. The center bar represents the mean of the data and error bars represent SD. Also see Supplementary Table 1. **d** Processivity of the BLM NTD truncation mutants compared to full-length BLM. Reactions contained 0.2 nM BLM (or the indicated BLM mutant) and 2 nM RPA but did not contain DNA2. The center bar represents the mean of the data and error bars represent SD. Also see Supplementary Table 1. **e** Kymographs showing examples of full-length 0.2 nM BLM or 0.2 mM BLM^Δ1–641^ and 2 nM RPA-GFP (green) in reactions with a 3′ ATTO565 (magenta) end-labeled DNA substrate. **f** Fraction of 3′ end looping events for each different BLM NTD deletion protein. The height of each bar represents the percentage DNA molecules that exhibit 3′ end looping and error bars represent 95% confidence intervals (CI) calculated from bootstrap analysis.

We constructed a series of truncation mutants to help identify which region of BLM might contribute to ssDNA loop formation (Fig. 4a). For these mutants, we focused initial efforts on generating a series of seven NTD (amino acid residues 1 to 641) truncations while leaving the helicase core, RQC, and HRDC domains intact (Fig. 4a).

Deletion of the entire NTD (GFP-BLM^Δ1–641^) yielded a truncated protein that was still efficiently targeted to the free DNA ends (74.7%, *N* = 112/150) and similar results were obtained for the other six NTD deletion proteins (Fig. 4b and Supplementary Fig. 4). In addition, all of the NTD truncation mutants underwent translocation, yielding velocity and processivity values that were comparable to, or, in some cases, significantly exceeded those obtained for full-length BLM (Fig. 4c, d and Supplementary Table 1). These effects were particularly apparent for GFP-BLM^Δ1–641^, which translocated 54% faster (54 ± 18 bp s^–1^ vs. 35 ± 17 bp s^–1^, *p* < 0.0001) and 131% further (28.0 ± 12.0 kb vs. 12.1 ± 6.1 kb, *p* < 0.0001) than full-length BLM in reactions without DNA2 (Fig. 4c, d and Supplementary Table 1). Interestingly, GFP-BLM^Δ1–641^ also had visibly dimmer GFP-BLM fluorescent signal at the ends of the DNA molecules, suggesting that full deletion of the NTD may have altered the oligomeric state of the DNA-bound BLM (Supplementary Fig. 4b, inset). Indeed, quantitation of the integrated signal intensity for full-length BLM and the other NTD truncation mutants revealed that the overall GFP signal intensity of GFP-BLM^Δ1–641^ complexes was 79.6% lower (*p* < 0.0001) than that of full-length BLM (Supplementary Fig. 5a). In addition, photobleaching step measurements confirmed that the majority (82.1%, *N* = 23/28) of the end-bound GFP-BLM^Δ1–641^ molecules exhibited single-step photobleaching behavior, suggesting that just one molecule of GFP-BLM^Δ1–641^ was bound (Supplementary Fig. 5b, c). These findings are consistent with prior reports indicating that the NTD is responsible for controlling BLM oligomerization^44–46^. None of the other NTD truncations showed such a large reduction in fluorescence signal intensity as was observed when the entire NTD was deleted (GFP-BLM^Δ1–641^), although two mutants (BLM^Δ236–333^ and BLM^Δ334–431^) showed ≥54% reduction in signal intensity (*p* < 0.0001) relative to full-length BLM, suggesting that multiple contacts within the NTD contribute to BLM oligomerization at DNA ends (Supplementary Fig. 5a).

### The BLM N-terminal domain contributes to DNA looping

We next asked whether the NTD deletion mutants could support DNA end looping. For this, we used GFP-tagged RPA to mark ssDNA generated by DNA end resection. It should be noted that the use of GFP–RPA precluded us from visualizing GFP-BLM but offered an advantage in allowing us to readily determine whether or not the 3′ ssDNA overhang was in an extended or a compacted conformation consistent with a loop-like structure (Fig. 4e). Interestingly, there was an 80% reduction in the propensity to form looped ssDNA ends during DNA end resection for the GFP-BLM^Δ1–641^ truncation mutant compared to full-length BLM (18.0 ± 15.8%, *N* = 117 vs. 91.4 ± 3.6%, *N* = 86; Fig. 4e, f). The other five NTD truncation mutants exhibited modest reductions in the percentage of looped ssDNA ends relative to full-length BLM, ranging from a 5.0% reduction for GFP-BLM^Δ134–235^ to 28.2% reduction for GFP-BLM^Δ334–431^, but these effects were not as striking as was observed for GFP-BLM^Δ1–641^ (Fig. 4f). We also noted that of all the BLM species analyzed, GFP-BLM^Δ1–641^, which is impaired in the ability to form ssDNA loops, translocated faster and farther than any of the other BLM constructs, including full-length BLM (Fig. 4c, d).

### The BLM C-terminal domain is necessary for DNA end recognition

**Fig. 5:**
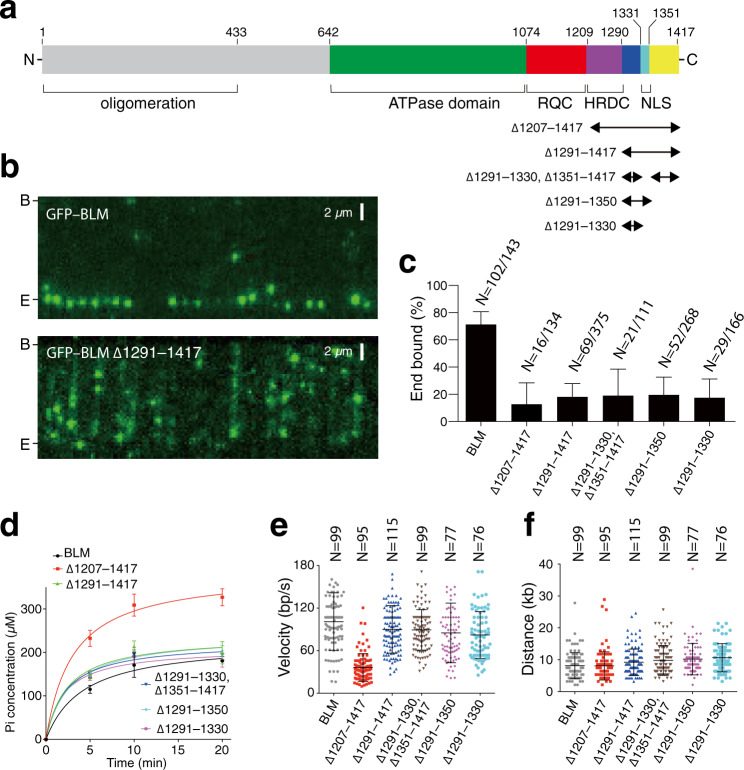
The CTD of BLM contributes to DNA end-binding activity. **a** Schematic showing the domain organization of BLM. Black arrowheads indicate the CTD truncation mutants analyzed in this study. **b** Wide-field TIRFM images showing full-length GFP-BLM (green) vs. GFP-BLM^Δ1291–1417^ bound to a single-tethered dsDNA curtain (unlabeled). Note, the upper panel (BLM) is reproduced from Fig. 1b for comparison. **c** Fraction of end-bound BLM complexes for each of the CTD deletion constructs compared to full-length BLM. The height of each bar represents the percentage end-bound BLM molecules and error bars represent 95% confidence intervals (CI) calculated from bootstrap analysis. Also see Supplementary Fig. 6. **d** ATP hydrolysis assays with the BLM CTD truncation mutants. All reactions contained 5 nM of the indicated BLM construct and M13 ssDNA (2 μM nucleotides). Data points represent the mean and error bars represent SD from three independent experiments. **e** Translocation velocity of BLM CTD truncation mutants bound to internal positions on the DNA. The center bar represents the mean of the data and error bars represent SD. Also see Supplementary Table 1. **f** Processivity of BLM CTD truncation mutants bound to internal positions on the DNA. The center bar represents the mean of the data and error bars represent SD. Also see Supplementary Table 1.

We next tested a series of C-terminal truncation mutants to assess their potential impact upon DNA end processing (Fig. 5a). The truncation mutant BLM^∆1207–1417^ lacks the entire HRDC domain and all of the remaining CTD amino acid residues (Fig. 5a). Interestingly, BLM^∆1207–1417^ could still bind dsDNA, but failed to selectively bind to the 30-nt 3′ ssDNA overhang in the dsDNA. Instead, this BLM mutant appeared to bind randomly along the length of the dsDNA (Fig. 5b, c and Supplementary Fig. 6a, b). Despite the fact that BLM^∆1207–1417^ hydrolyzed ATP at a higher rate than full-length BLM (Fig. 5d–f and Supplementary Table 1), a 64% reduction in translocation velocity was measured for this mutant (36 ± 20 bp s^–1^ vs. 101 ± 35 bp s^–1^ for full-length BLM)^17^. This latter finding is consistent with prior reports indicating that deletion of the HRDC partially decoupled ATP hydrolysis from duplex DNA unwinding, resulting in nonproductive ATP hydrolysis cycles^45,47^.

Next, we tested BLM^∆1290–1417^, which lacks the BLM C-terminal region but retains the HRDC domain (Fig. 5a). Surprisingly, BLM^∆1290–1417^ also lost the ability to selectively bind to DNA ends, and instead associated with random locations along the dsDNA molecule (Fig. 5b and Supplementary Fig. 6). Unlike BLM^∆1207–1417^, which lacks the HRDC domain, the BLM^∆1290–1417^ truncation mutant had a similar ATPase activity compared to full-length BLM (Fig. 5d). It could also translocate on dsDNA even when it was not bound to a DNA end, as we had previously shown for full-length BLM^17^, and yielded velocity and processivity values comparable to those determined for full-length BLM (Fig. 5e, f and Supplementary Table 1). These results indicate that the HRDC domain plays a role in coupling ATP hydrolysis to DNA translocation. Importantly, our finding that BLM^∆1290–1417^ retains DNA translocase activity but fails to associate with DNA ends, indicates that CTD amino acid residues beyond the HRDC domain are necessary for BLM recruitment to DNA ends.

We next tested additional truncation mutants to help further define the BLM CTD region responsible for DNA end-binding activity (Fig. 5a). The results revealed that the CTD domain conferring DNA end-binding activity could be ascribed to the region encompassing amino acid resides 1291 to 1330 (Fig. 5a–e and Supplementary Fig. 6). We note that this tract of 40 amino acid residues is moderately conserved among BLM helicases from different organisms but is not found in other RecQ helicase family members, and it has been previously implicated as necessary for the ATP-independent ssDNA annealing activity of BLM^28,48^. Together, our results suggest that BLM C-terminal region encompassing amino acid residues 1291–1330 is required for efficiently targeting BLM helicase to DNA ends in vitro.

### Biochemical effects of BLM mutants

We next sought to further characterize the properties of some of the BLM mutants in bulk biochemical assays, focusing efforts on: (1) BLM^∆1–641^, which exhibited an altered oligomeric state and loss of ssDNA looping in the DNA curtain assays, but retained translocation activity; and (2) BLM^∆1290–1330^ which exhibited a complete loss of end-binding specificity but retained DNA translocase activity in the DNA curtain assays.

**Fig. 6:**
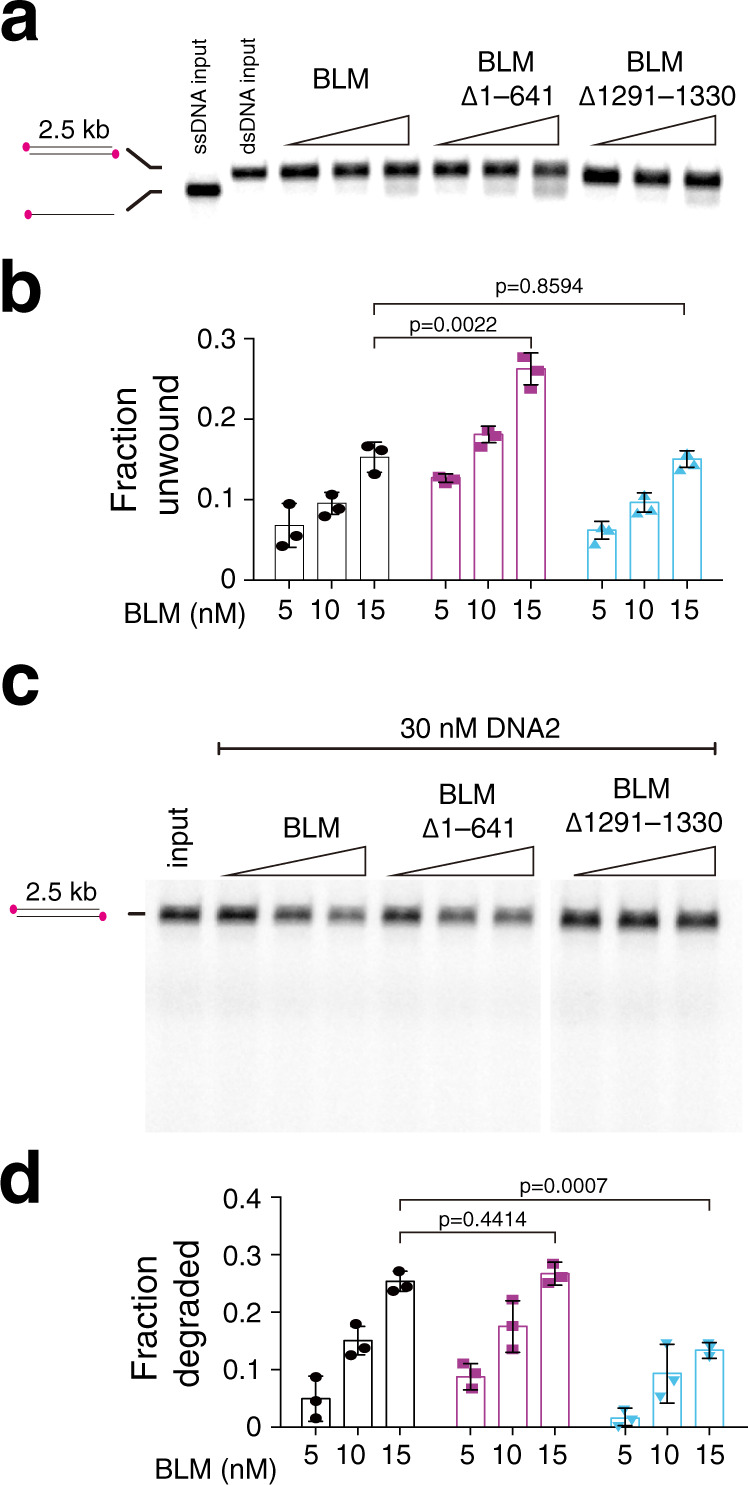
Bulk biochemical analysis of BLM^Δ1–641^ and BLM^Δ1291–1330^. **a** DNA unwinding assay with a 2.5 kb ATTO532N end-labeled substrate in assays comparing 2.5, 5, and 10 nM GFP-tagged full-length BLM with BLM^Δ1–641^ and BLM^Δ1291–1330^ in the presence of 200 nM RPA. **b** Quantitation of the DNA unwinding assay showing the fraction of unwound DNA product. The height of the bars represents the mean and error bars represent SD calculated from three separate reactions. **c** DNA degradation assay with a 2.5 kb ATTO532N end-labeled substrate in assays comparing 2.5, 5, and 10 nM full-length BLM with BLM^Δ1–641^ and BLM^Δ1291–1330^ in the presence of 30 nM DNA2 and 200 nM RPA. **d** DNA degradation assay quantitation showing the fraction of degraded DNA product. The height of the bars represents the mean and error bars represent SD calculated from three separate reactions.

We began with bulk biochemical assays using a labeled 2.5 kb dsDNA fragment to monitor DNA unwinding and resection^9^. The NTD truncation mutant BLM^∆1–641^ had a 72% increase in dsDNA unwinding ability compared to full-length BLM, consistent with its increased velocity and processivity in the single-molecule assays (Fig. 6a, b). Interestingly, BLM^∆1–641^ supported DNA2-dependent dsDNA resection at a level comparable to full-length BLM (Fig. 6c, d). The observation that resection activity was not elevated in assays with BLM^∆1–641^ and DNA2, despite the finding that BLM^∆1–641^ has enhanced translocase properties in single-molecule assays and greater DNA unwinding activity in bulk assays, suggests that DNA cleavage by DNA2 may be a rate limit step in these reactions. Together with the single-molecule data presented above, these biochemical analyses suggest that the reduced oligomerization and loss of ssDNA loop formation observed for BLM^∆1–641^ have little or no impact on DNA2-dependent DNA end resection in vitro.

Interestingly, BLM^∆1291–1330^ exhibited similar dsDNA unwinding activity compared to full-length BLM, even though it lost the ability to recognize DNA ends in the single-molecule assays (Fig. 6b). However, BLM^∆1291–1330^ was impaired for DNA end resection with DNA2, yielding a 47% reduction (*p* = 7 × 10^–4^) relative to full-length BLM (Fig. 6c, d). This reduction in DNA resection activity paralleled the loss of end-binding specificity for this CTD truncation mutant as observed in the DNA curtain assays (Supplementary Fig. 6f).

### Cellular effects of BLM mutants

**Fig. 7:**
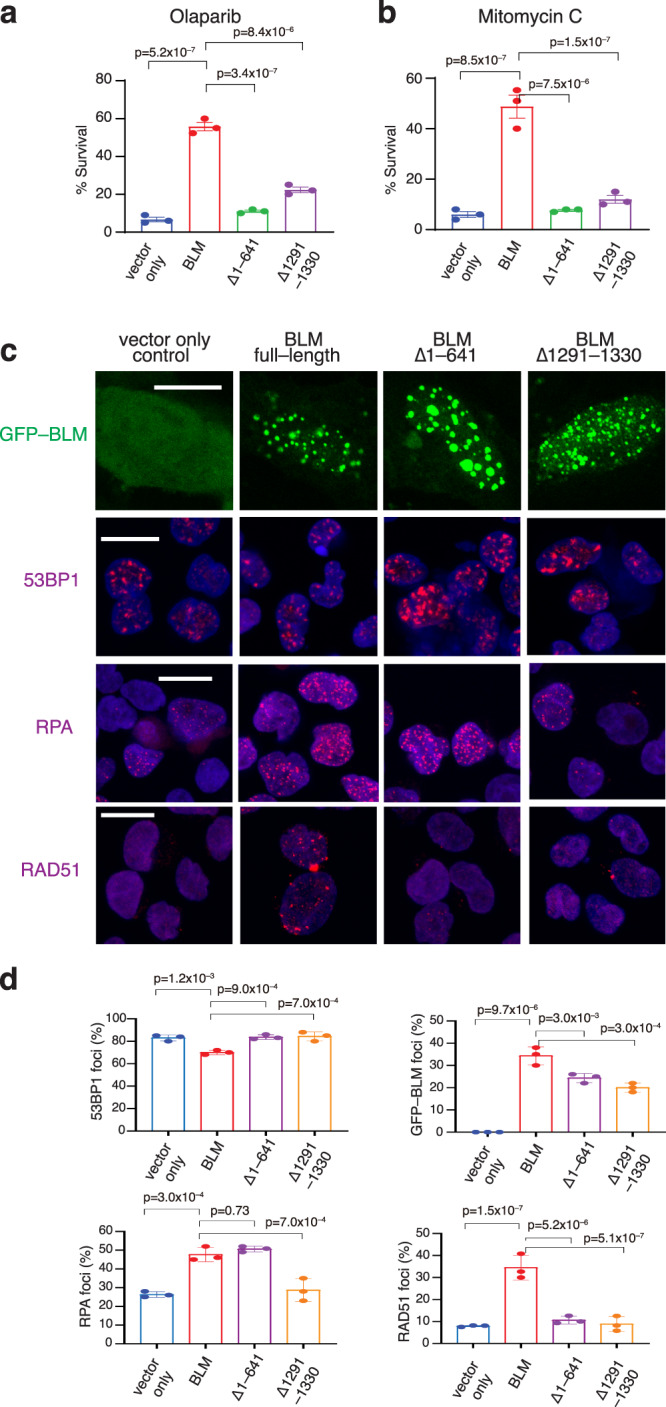
DNA repair foci defects in cells expressing BLM^Δ1–641^ and BLM^Δ1291–1330^. **a** Clonogenic survival assays for U2OS cells following double siRNA knockdown of BLM and EXO1 expressing either no BLM (vector only control), full-length GFP-BLM, GFP-BLM^Δ1–641^ or GFP-BLM^Δ1291–1330^, as indicated, after treatment with 17 nM Olaparib (the IC50 for U2OS cells expressing full-length GFP-tagged BLM). Data points represent the mean ± SEM of three independent experiments. **b** Clonogenic survival assays (as above) following treatment with 12 nM (the IC50 for U2OS cells expressing full-length GFP-tagged BLM) Mitomycin C. Data points represent the mean ± SEM of three independent experiments. **c** Cells were either visualized for GFP-BLM foci or stained with DAPI and immune-stained with antisera against 53BP1, RPA or RAD51, as indicated, 4 h after exposure to 8 Gy ionizing radiation. Scale bar = 15 µm. **d** Quantitation of 53BP1, GFP-BLM, RPA and RAD51 foci. Data points represent the fraction (%) of cells (mean ± SEM, *N* = three independent experiments) with greater than five foci per cell (≥300 cells were analyzed per condition).

Next, we conducted studies to investigate the cellular effects of BLM NTD and CTD truncations, again focusing efforts on the two mutants BLM^∆1–641^ and BLM^∆1291–1330^ that were examined in depth in vitro. U2OS cells were transfected with either a vector only control or a plasmid encoding the GFP-tagged BLM constructs (full-length BLM, BLM^∆1–641^ or BLM^∆1291–1330^) followed by siRNA knockdown of endogenous BLM and EXO1 expression (Supplementary Fig. 7a, b). Cells expressing either GFP-BLM^∆1–641^ or GFP-BLM^∆1291–1330^ exhibited significantly reduced colony formation in the presence of the PARP inhibitor Olaparib or the DNA alkylating reagent Mitomycin C (MMC) compared to cells expressing full-length GFP-BLM (Fig. 7a, b and Supplementary Fig. 7c, d). These findings revealed that cells expressing GFP-BLM^∆1–641^ or GFP-BLM^∆1291–1330^ are likely impaired for BLM-dependent DNA repair.

To help define the underlying molecular defects of cell expressing the BLM mutants, we performed immunofluorescence assays to examine BLM, 53BP1, RPA and RAD51 foci upon exposure to 8 Gy ionizing radiation (IR). 53BP1 is an early marker of DSBs, and comparable levels of IR-induced 53BP1 foci were observed among cell types that express the various BLM species (Fig. 7c, d). GFP-BLM^∆1–641^ and GFP-BLM^∆1291–1330^ both formed IR-induced foci, albeit to a lesser extent than full-length GFP-BLM, with GFP-BLM^∆1291–1330^ exhibiting a slightly greater defect (Fig. 7c, d). These data suggested that the BLM mutant proteins are recruited to IR-induced DNA damage. Notably, cells expressing GFP-BLM^∆1291–1330^ were defective in the assembly of both RPA and RAD51 foci, suggesting that GFP-BLM^∆1291–1330^ was unable to support DNA end resection (Fig. 7c, d). In sharp contrast, even though GFP-BLM^∆1–641^ supported IR-induced RPA focus formation at levels comparable to full-length GFP-BLM, this NTD deletion mutant had a strong defect in the assembly of RAD51 foci (Fig. 7c, d). Taken together, these findings suggest that the survival defect of cells expressing GFP-BLM^∆1291–1330^ likely stems from a DNA end resection defect, whereas the DNA damage sensitivity of cells expressing GFP-BLM^∆1–641^ is likely caused by an inability to assemble the RAD51 presynaptic complex after DNA end resection.

**Fig. 8:**
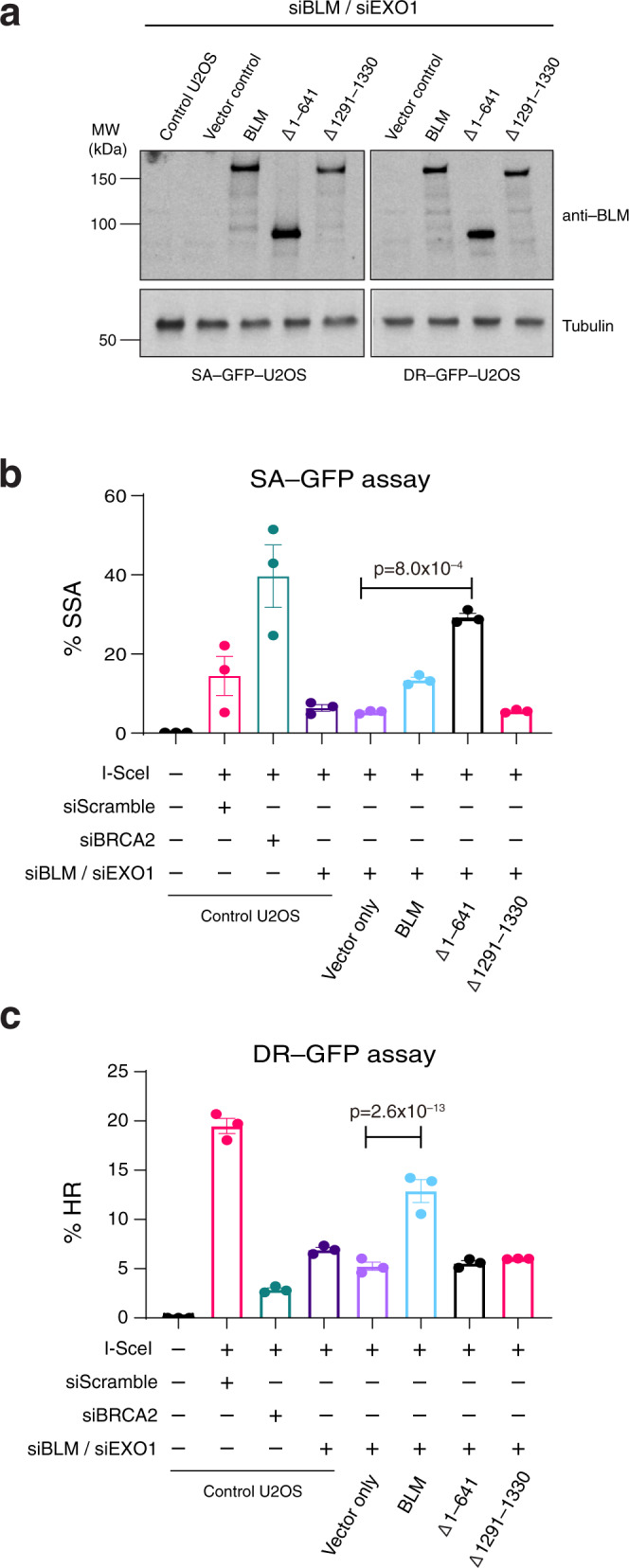
Cell-based reporter assays for SSA and HR in cells expressing BLM^Δ1–641^ or BLM^Δ1291–1330^. **a** Western blots of extracts from U2OS (SA-GFP and DR-GFP) cells depleted for BLM and EXO1 were reconstituted with the indicated genotypes and immune-stained with anti-BLM and anti-Tubulin antisera, as indicated. **b** Bar graph showing results for the SA-GFP reporter assay in U2OS cells with the indicated genotypes; data points represent %SSA. **c** Bar graph showing results for the DR-GFP reporter assay in U2OS cells with the indicated genotypes; data points represent %HR. In **b** and **c** data points reflect the mean ± SEM for *N* = three independent experiments.

Next, we employed cell-based DR-GFP and SA-GFP reporter assays to test for the fraction of I-SceI induced DSBs that were repaired by either HR or single-strand annealing (SSA)^49^. For these assays, U2OS cells depleted for endogenous BLM and EXO1 expression by siRNA knockdown and harboring integrated copies of either the DR-GFP or the SA-GFP cassette were transformed with BLM expression constructs (vector only, full-length BLM, BLM^Δ1–641^ and BLM^Δ1291–1330^) and a plasmid encoding I-SceI (Fig. 8a). Control experiments confirmed that siRNA knockdown of BRCA2 led to an increase in SSA-mediated repair (Fig. 8b) and also caused a corresponding loss of HR-mediated repair, as expected (Fig. 8c). Notably, cells expressing the BLM NTD truncation mutant BLM^Δ1–641^ exhibited significantly higher levels of SSA compared to WT-BLM (Fig. 8b). In contrast, cells expressing BLM^Δ1291–1330^ were defective for SSA, yielding SSA levels comparable to a vector only control (Fig. 8b). Moreover, cells expressing either BLM^Δ1–641^ or BLM^Δ1291–1330^ were both defective for HR-mediated repair compared to WT-BLM complemented cells (Fig. 8c). To further validate these results, we quantified end resection at DSBs using qPCR to detect production of ssDNA after expression of the endonuclease *Asi*SI in U2OS cells depleted of endogenous BLM and EXO1 and transformed with full-length BLM, BLM^Δ1–641^ or BLM^Δ1291–1330^ expression constructs (Supplementary Fig. 8a)^50^. We found that depletion of endogenous BLM and EXO1 resulted in a large decrease in ssDNA levels that was partially restored by ectopic expression of full-length WT-BLM or BLM^Δ1–641^ but was not restored by BLM ^Δ1291–1330^ (Supplementary Fig. 8b). Taken together, these findings suggested that BLM^Δ1–641^ was able to resect DSB ends, but these ends were channeled into an SSA-mediated repair pathway instead of HR likely due to a defect in RAD51 filament formation, whereas BLM ^Δ1291–1330^ was unable to process DNA ends for repair by either SSA or HR.

## Discussion

Here, we have examined the impact of NTD and CTD truncation mutations on the biochemical attributes of BLM and on DNA end resection in conjunction with DNA2 and RPA. Our data suggest that DNA end resection coincides with the formation of a large ssDNA loop and suggests that productive end resection may involve multimeric forms of the BLM helicase. Our findings have important implications for understanding the mechanisms of DNA end resection in metazoans.

### Model for DNA end resection by human BLM–DNA2

Our data help to define the molecular mechanisms underlying BLM–DNA2-dependent DNA end resection and the role of BLM in downstream steps of HR. Our results suggest that initial DNA end recognition by BLM results in the formation of a stable end-bound complex that becomes activated for end resection upon addition of DNA2, RPA or both. The involvement of DNA2 and RPA in activating end-bound BLM for translocation is very similar to what we have previously reported for *S. cerevisiae Sgs1*, which binds DNA ends in an inactive apo configuration that can be activated by DNA2 or RPA^35^. Interestingly, even with both DNA2 and RPA present, end-bound BLM still translocated much more slowly compared to BLM initially bound to an internal region of dsDNA (~10–15 bp s^–1^ vs. ~100 bp s^–1^, in our assays)^17,39,40^. In addition, we also find that the presence of DNA2 can greatly slow the translocation velocity of end-bound BLM (~10–15 bp s^–1^ vs. ~40 bp s^–1^), as has been previously observed^40^. These results suggest that the translocation characteristics of BLM are drastically different during DNA end processing compared to reactions in which BLM is simply unwinding a dsDNA molecule from an internal location, consistent with the premise that the structure of the bound DNA, together with the presence of DNA2, have important regulatory impacts upon BLM.

We also find that end resection coincides with the formation of a large ssDNA loop or loop-like structure that is coated with RPA. BLM itself is primarily responsible for DNA loop formation, presumably by maintaining contact with the 3′ ssDNA end, or perhaps by maintaining contact with RPA bound at or near the 3′ ssDNA end (see below)^23^, as the resection machinery progresses along the dsDNA. Such a mechanism for ssDNA loop formation by human BLM bears some similarity to the DNA end-binding activity of Rep, NS3, PcrA, Srs2, Pif1 and several other helicases, which enables the helicase to undergo repetitive shuttling behavior on DNA fragments as revealed in smFRET studies^51–54^.

One can envision a number of possible benefits of coupling DNA end resection to ssDNA loop formation. For instance, formation of an ssDNA loop may help to protect the 3′ ssDNA end to ensure that it is not degraded by other cellular nucleases. Formation of a ssDNA loop may also help to coordinate other downstream events such as the recruitment of protein components during formation of the presynaptic complex, including proteins such as BRCA1–BARD1, BRCA2, RAD51 and RAD52. A model in which end resection is somehow linked to presynaptic complex assembly is supported by experiments with the mutant BLM^∆1–641^, which is proficient for DNA end resection in vitro and in vivo but does not support formation of RAD51 foci in cells exposed to IR. These findings also imply that BLM itself may play a more direct role in loading RAD51 onto newly resected ssDNA than previously appreciated and suggests the possibility that end resection and presynaptic complex formation may be temporally coordinated through the actions of BLM.

### Contributions of the BLM NTD to DNA end resection

Our findings demonstrate the NTD encompassing amino acid residues 1–641 exerts a clear impact upon BLM oligomerization and ssDNA loop formation during DNA end resection. It seems possible that BLM oligomers possess a multiplicity of DNA-binding domains to allow for stable interactions with the newly generated ssDNA even as the end resection machinery travels along the DNA substrate. For example, if one BLM protomer drives ATP-dependent translocation, then one or more of the other protomers within the oligomeric complex may remain stably bound to the end of the unwound ssDNA without undergoing translocation.

Interestingly, BLM^∆1–641^ translocates on DNA at a faster rate on dsDNA and with higher processivity than full-length BLM, suggesting that assembly of higher order BLM oligomers affects the helicase activity of BLM negatively. Moreover, our data show that BLM^∆1–641^ supports end resection in vivo as evidenced by the appearance of RPA foci. In sharp contrast, BLM^∆1–641^ fails to support RAD51 focus formation in vivo and I-SceI induced DSBs are channeled through SSA-mediated DNA repair instead of HR, consistent with reduced RAD51 filament formation in cells expressing BLM^∆1–641^. As we have speculated above, the NTD of BLM may be somehow involved in loading RAD51 onto processed DNA breaks. In vitro DNA resection assays using BLM and EXO1 have also led to the suggestion that RAD51 loading onto the resected DNA ends may be facilitated through protein–protein interactions between RAD51 and BLM^55^. Notably, BLM and RAD51 co-immunoprecipitates from cellular extracts and a combination of two-hybrid and Far Western analyses mapped RAD51 interaction regions to the N-terminus (amino acid residues 1–212) and C-terminus (amino acid residues 1317–1417) of BLM^56^. The mutant BLM^Δ134–235^ supports DNA end looping (Fig. 4f) and overlaps with the N-terminal region implicated in interactions with RAD51. Therefore, we examined RPA and RAD51 foci formation in cells expressing BLM^Δ134–235^ to determine whether this NTD truncation might serve as a separation-of-function mutant that supported DNA looping but was defective for RAD51 loading (Supplementary Fig. 9). Interestingly, there was no significant changes in RPA foci formation for cells expression BLM^Δ134–235^ compared to WT-BLM, however, there was a moderate decrease in RAD51 foci (20.5% decrease, *p* < 0.05; Supplementary Fig. 9). Thus BLM^Δ134–235^ is moderately deficient for RAD51 loading yet still capable of DNA looping. Given that BLM can interact with RAD51 through both its NTD and CTD^56^, it may be insufficient to eliminate just one of the two interactions regions to fully disrupt in vivo RAD51 loading. Unfortunately, the second region implicated in RAD51 interaction overlaps with the CTD region which we find to be essential for BLM targeting to DNA ends. More detailed mapping of the RAD51 interaction domain may facilitate identification of BLM mutants in the N- and C-terminal regions which are disrupted for RAD51 interactions, but still capable of targeting DNA ends.

As indicated above, BLM may help mediate DNA loop formation by maintaining contact with the 3′ ssDNA end or perhaps by binding to RPA bound at or near the 3′ ssDNA end, as the resection machinery progresses along the dsDNA. Interestingly, early studies revealed that BLM physically interacts with RPA and this interaction domain maps to the BLM N-terminal region encompassing amino acid residues 1 through 477^57,58^. More recent studies have revealed two separate RPA interaction motifs within the N-terminus of BLM encompassing amino acid residues 150–163 and 551–565^23^. Interestingly, two of our BLM truncation mutants should independently eliminate each of these two motifs (BLM^∆134–235^ and BLM;^∆532–641^ Fig. 4a), nevertheless, both mutants retain 3′ end DNA looping activity (Fig. 4f). However, if two distinct BLM–RPA interactions can independently contribute to DNA loop formation, then the existence of these two separate RPA interaction motifs could explain why we only see looping defects for the BLM^∆1–641^ truncation mutant. It is possible that further analysis of the BLM–RPA, BLM–RAD51, and BLM–ssDNA interactions may help to disentangle the contributions of these different interactions to DNA looping and presynaptic complex formation.

### The oligomeric state of end-bound BLM

BLM can oligomerize in solution but dissociates into monomers upon addition of ATP^44,59–61^. A conserved helical bundle (amino acid residues 362–414) is involved in BLM dimerization and possibly hexamer formation^44^. Surprisingly, we have found that when amino acid residues spanning this region are deleted (i.e. BLM^Δ334–431^), we could still see some evidence for BLM oligomerization at DNA ends, albeit to a significantly lesser extent than is observed for full-length BLM. Indeed, all of our BLM constructs, with the exception of BLM^Δ1–641^, show evidence for some oligomerization at the DNA ends. Interestingly, by electron microscopy, BLM has been shown to exhibit a marked tendency to oligomerize through protein–protein interactions on DNA substrates mimicking D-loops with a 5′ invading end^59^. Comparable measurements have not been made on DNA substrates mimicking end processing intermediates. Thus, our data may reflect some propensity of multiple BLM monomers to interact with one another while bound to DNA ends.

### Contributions of the BLM CTD to DNA end recognition

The function of the HRDC domain located near the BLM CTD has been somewhat enigmatic^62^. Of the five human RECQ helicases, BLM and WRN possess this domain, whereas RECQ1, RECQ4 and RECQ5 do not^26,27,62^. The HRDC is necessary for targeting BLM to sites of DNA damage in vivo^30,31^, but there are conflicting reports regarding its involvement in vitro in DNA-binding^63,64^. The crystal structure of BLM (residues 640–1298) bound to DNA shows that the HRDC domain is ~28 Å away from the DNA^47^. Thus, one interpretation of the cellular data is that HRDC helps target BLM to DNA damage not through direct contacts with DNA, but instead through protein–protein contacts with some other DNA repair factor. We find that deletion of the entire BLM CTD from amino acids 1207 to 1417 (i.e. BLM^∆1207–1417^), which includes the HRDC, abolishes DNA end recognition in vitro, but this mutant protein is still able to bind internal positions along the dsDNA. Interestingly, even though BLM^∆1207–1417^ exhibits a marked increase in ATPase activity, its translocase attribute is significantly impaired (54% lower) compared to full-length BLM. Together, these results suggest that removal of the HDRC domain partially decouples BLM ATP hydrolysis from DNA translocation, resulting in futile cycles of ATP hydrolysis that do not result in protein movement along the DNA, as has been suggested previously^47^.

We have also identified a conserved peptide sequence encompassing amino acid residues 1290 to 1330 that is essential for BLM recruitment to DNA ends. This peptide sequence flanks the HRDC domain but is not a part of the HRDC domain itself. Deletion of these amino acid residues (i.e. BLM^∆1291–1330^) abolishes DNA end recognition in vitro, but unlike the removal of the entire HRDC domain, deleting this short peptide has little or no impact on BLM ATPase or translocase activity. Remarkably, despite its inability to target DNA ends in vitro, BLM^∆1291–1330^ does form IR-dependent foci in cells, suggesting that it is recruited to DNA damage. However, RPA and RAD51 focus formation was severely compromised in cells expressing BLM^∆1291–1330^, indicating that this mutant was unable to support normal DNA end resection in vivo. Thus, while BLM^∆1291–1330^ can be recruited to DNA damage through specific protein–protein interactions with other repair factors, perhaps involving the HRDC domain^31^, it is unable to productively initiate DNA processing due to its DNA end recognition defect. We favor a model in which amino acid residues 1290 to 1330, perhaps together with the flanking HRDC domain^65^, may exert an allosteric or structural influence over the RQC domain, the helicase core domain, or both domains, which modulates their ability to specifically recognize DNA ends.

### Potential similarities to other end resection systems

We have previously reported our DNA end resection studies using the *Saccharomyces cerevisiae* proteins Sgs1, Dna2, Top3–Rmi1 and RPA^35^. Strikingly, RPA-mCherry used in our work accumulated on ssDNA generated during DNA end resection. Moreover, the ssDNA ends remained highly compacted and tracked with GFP–Sgs1 as it progressed along DNA, indicating that the resulting ssDNA may have also existed in a loop-like configuration. Interestingly, a recent report has suggested that a ssDNA loop involving the 5′ terminated strand may also form due to the differential velocities of Sgs1 and DNA2^66^, similar to bacterial RecBCD (discussed below), although we note that this model for ssDNA loop formation is distinct from our present results with the human enzymes, because we show that DNA2 is not necessary for ssDNA loop formation and the 3′ end of the ssDNA remains associated with the resection machinery as it travels along the dsDNA.

Interestingly, our model for end resection by BLM–DNA2 bears some resemblance to what takes place during DNA end resection by the *E. coli* RecBCD and *B. subtilis* AddAB helicase/nuclease complexes, although the overall mechanisms of the human and these bacterial end resection machineries are quite distinct^67–72^. As with the BLM–DNA2 system, it is not yet clear whether ssDNA loop during end resection in bacteria is an essential part of DNA repair in bacteria. However, protein–protein interactions between the RecB subunit of RecBCD and RecA facilitate RecA loading onto the ssDNA strand generated during end resection^73–77^, although it is as yet unknown whether RecA loading might be influenced by or coupled to ssDNA loop formation. Given the above, it will be of great interest to examine the potential relationship between ssDNA loop formation during DNA end resection and presynaptic complex formation mediated by BLM–DNA2, RecBCD and AddAB.

Our data reveal mechanistic insights into BLM as it fulfills its role in DNA end processing in conjunction with DNA2 and RPA. We note that the activity of BLM in DNA end resection is also influenced by other proteins, including the TOP3–RMI complex, the MRE11–RAD50–NBS1 complex, and CtIP^9,41,78–81^. Moreover, within the cellular setting, DNA end processing is likely to be intimately coupled to assembly of the RAD51–ssDNA presynaptic complex, and our data suggest that BLM itself may help couple end processing and RAD51 loading. The assays presented here offer a valuable tool to define the impact of accessory factors on DNA end processing and presynaptic complex assembly.

## Methods

### Plasmid construction

BLM truncation mutants were generated through divergent PCR cloning using GFP-BLM–pYES2 as a template^17^. For BLM transient transfection in siBLM/siEXO1 U2OS cells, the BLM constructs were subcloned into the pBI–EGFP vector using Gibson assembly.

### Protein expression

RPA, RPA-mCherry, RPA-GFP, DNA2, GFP-BLM, and GFP-BLM (K695A) were purified as previously described^17,82,83^. GFP–BLM truncation mutants were purified similarly to full-length GFP–BLM, with some minor modifications. In brief, the GFP–BLM mutant expression plasmids were transformed into a protease-deficient yeast expression strain (JEL–1). Cells were grown in six liters of basic medium minus uracil (0.17% yeast nitrogen base, 0.5% ammonium sulfate, 2% sodium lactate, 3% glycerol, 0.87 g/l amino acid mix without uracil) to 1.0 OD600 at 30 °C and then induced overnight at 25 °C with the addition of 2% galactose. Cells were harvested by centrifugation and lysed by vortexing in 40 ml cell lysis buffer (50 mM Tris-HCl [pH 7.0], 1 M NaCl, and 10% glycerol, 1 mM TCEP, protease inhibitor cocktail (Roche, Cat. No.: 05892988001), 2 mM EDTA) with the same volume of glass beads (425–600 µm; Sigma, Cat. No.: G8772) at 4 °C. All cell lysates were precipitated with 20% ammonium sulfate (10 g per 50 ml supernatant). After 1 h, the protein pellets were recovered by centrifugation at 13,000 × *g* at 4 °C for 10 min. The protein pellets were then dissolved in 20 ml cell lysis buffer without EDTA plus 25 mM imidazole. These protein resuspensions were passed through a 0.45 µm filter (Millex; Cat No.: SLHV033RS) to remove any undissolved precipitate. The protein solutions were then purified using Ni-NTA (Qiagen) resin and eluted with an imidazole step gradient using successive washes of buffer containing 20, 30, 40, 50, 60, 70, 80, 100, 120, 140, 160, 180, 200 and 250 mM imidazole and peak fractions containing GFP–BLM were identified by SDS-PAGE and Coomassie staining. The purified GFP–BLM fractions were combined aliquoted and stored at –80 °C.

### DNA substrates

The λ-DNA substrates were prepared as previously described^17^. For dsDNA with a 30-nt 3′-end tail, λ-DNA (NEB, Cat. No. N3011S) was annealed to a biotinylated oligo (5′-Phos-AGG TCG CCG CCC-3′BioTEG), and the second end of the λ–DNA was annealed to the oligonucleotide with the following sequence: 5′-Phos-GGG CGG CGA CCT TTT TTT TTT TTT TTT TTT TTT TTT TTT TTT-3′ to yield a dsDNA substrate with a 3′ 30-nt poly(dT) overhang, as previously described^35^. For dsDNA with the 30-nt 3′ end tail end-labeled with ATTO565, the λ-DNA was annealed the biotinylated oligo as described above, and the second end of the λ–DNA was annealed to the oligonucleotide with the following sequence: 5′-pGGG CGG CGA CCT TTT TTT TTT TTT TTT TTT TTT TTT TTT TTT-ATTO565N-3′. For dsDNA with a 30-nt 5′ end tail labeled with ATTO565, λ–DNA was annealed to the biotinylated oligo, and the second end of the λ–DNA was annealed to two oligonucleotides with the following sequence: 5′-ATTO565N-AGT GTC GTG CCG G-3′ and 5′-Phos-GGG CGG CGA CCT CCG GCA CGA CAC TTT TTT TTT TTT TTT TTT TTT TTT TTT TTT T-3′. All substrates were ligated overnight using T4 DNA ligase (NEB, Cat. No. M0202S) and purified by PEG precipitation, as described^17^.

The 2.5 kb dsDNA substrate used for the bulk biochemical assays was a PCR amplified fragment of pFastBac–HTB generated using PCR primers with the following sequence: 5′-ATTO532N-ATC ACT GAT ATC GCC TAG G-3′ and 5′-ATTO532N-ACC AAT GCT TAA TCA GTG AGG-3′. After the PCR reaction, the substrates were purified by electrophoresis in a 0.8% agarose gel stained with SYBR Safe and purified using Wizard SV gel clean-up system (Promega, Cat. No. A9281)

### Bulk biochemical helicase and nuclease assays

BLM helicase and BLM–DNA2 nuclease assays were performed in BLM helicase buffer (20 mM Tris-HCl [pH 7.5], 1 mM MgCl_2_, 2 mM ATP, 1 mM DTT, 0.2 mg/ml BSA, 10 mM creatine phosphate, 0.05 mg/ml creatine kinase, and 200 nM RPA) with 1.5 nM DNA ends (2.5 kb dsDNA substrate) at 37 °C for 15 min. Reactions were initiated by adding 2.5, 5, or 10 nM BLM with or without 30 nM DNA2 and stopped by adding SDS to 0.2% and protease K to 0.25 mg/mL. Then reaction mixtures were incubated for another 5 min at 37 °C. The resulting samples were loaded onto a 1% agarose gel 1xTAE buffer. Gels were scanned using a Typhoon imager (GE Healthcare) and the intensity of the dsDNA and ssDNA bands was quantified using ImageJ.

### ATPase assays

ATPase assays for BLM variants were performed in BLM buffer (20 mM Tris-HCl [pH 7.5], 1 mM MgCl_2_, 2 mM ATP, 1 mM DTT, 0.2 mg/ml BSA) at 37 °C with M13 ssDNA (2 μM nucleotides, NEB, Cat. No. N4040S). Reactions were initiated by the addition of 5 nM BLM variants, as indicated. Aliquots were removed at the indicated time points and quenched by addition of 50 mM EDTA. The quenched reactions were quantified by the ATPase/GTPase Activity Assay Kit as per the manufacturer’s instructions (Sigma, Cat. No. MAK113).

### Single-molecule dsDNA curtain assays

All experiments were conducted with a prism-type total internal reflection fluorescence microscope (Nikon) equipped with a 488-nm laser (Coherent Sapphire, 200 mW), a 561-nm laser (Coherent Sapphire, 200 mW), and two Andor iXon EMCCD cameras^84^. Flowcells and dsDNA curtains were prepared as previously described^35,36^. In brief, lipid bilayers were prepared with 91.5% 1,2-dioleoyl-sn-glycero-3-phosphocholine (DOPC; Avanti Polar Lipids, Cat. No. 850375C), 0.5% biotinylated-1-palmitoyl-2-oleoyl-sn-glycero-3-phosphoethanolamine (Biotin-PE; Avanti Polar Lipids, Cat. No. 860562C), and 8% mPEG 2000-1,2-dioleoyl-sn-glycero-3-phosphoethanolamine-N-[4-(p-(cysarginyl-glycylaspartate-maleimidomethyl) cyclohexane-carboxamide] (mPEG-DOPE; Avanti Polar Lipids, Cat. No. 880130C) and deposited onto the surface of flowcell sample chamber, as described^35,36,84^. The biotinylated λ–DNA substrates were then injected into the sample chamber and attached to the bilayer through a biotin-streptavidin linkage. The flowcell was then connected to a microfluidic system and sample delivery was controlled using a syringe pump (Kd Scientific)^84^. Biotinylated dsDNA substrates were aligned at the barriers by application of flow in BLM buffer (20 mM Tris-HCl [pH 7.5], 1 mM MgCl_2_, 2 mM ATP, 1 mM DTT, 0.2 mg/ml BSA, ±0.5 nM YOYO-1 (Thermo Fisher Scientific, Cat. No. Y3601), as indicated) at 37 °C with a flow rate of 0.15 ml/min.

For experiments, images were collected at 1 frame per 20 or 30 s with 0.1 s integration time using two EMCCD cameras with a custom-built shuttering system to avoid signal bleed-through during image acquisition^84^. Data acquisition was controlled using NIS-Elements Version 5.1 (Nikon). Image acquisition was started immediately before the protein injections and the illumination lasers were shuttered between each acquired image to minimize photobleaching. GFP–BLM or the GFP–BLM truncation mutants (0.2 nM) in BLM buffer were injected into the flow cell through a 150 µl loop. Then, 2 ml BLM washing buffer (20 mM Tris-HCl [pH 7.5], 1 mM MgCl_2_, 2 mM ATP, 1 mM DTT, 0.2 mg/ml BSA, and 100 mM NaCl) was injected into the sample chamber with a flow rate of 1 ml/min. Reactions were initiated by injection of the indicated proteins (0.2 nM DNA2, 2 nM RPA, or 2 nM RPA-mCherry, or 2 nM RPA-GFP) in BLM buffer at 0.25 ml/min. For experiments using both GFP–BLM and RPA-GFP, the GFP–BLM was photobleached prior to the injection of RPA-GFP.

### Single-molecule data analysis

Single-molecule image analysis was performed using Open-source image processing software ImageJ (Version: 2.0.0-rc-59/1.51k, http://imagej.net/Contributors). Raw TIFF images were imported as image stacks into ImageJ and images were corrected for drift using the StackReg function in ImageJ^84^. Kymographs were then generated from the corrected image stacks by defining a 1-pixel wide region of interest encompassing individual dsDNA molecules, and these kymographs were used for analysis of GFP–BLM processivity, velocity and binding distributions as previously described^17,35^.

### Cell culture

U2OS cells were grown in Dulbecco’s Modified Eagle Medium medium (Thermo Fisher, Cat. No.: 10569010) supplemented with 10% V/V Fetal Bovine Serum (Fisher Scientific, Cat. No.: Corning™ 35011CV) and 1% V/V penicillin-streptomycin antibiotic (Thermo Fisher, Cat. No.: 15140122).

### Plasmid and siRNA transfections

pBI–MCS–EGFP (a generous gift from Bert Vogelstein (Addgene, plasmid #16542)^85^ containing WT-BLM, BLM^Δ1–641^ and BLM^Δ1291–1330^ were used to transfect the U2OS cells in a 6 well using Lipofectamine 3000 Transfection Reagent (Invitrogen, Cat. No.: L3000015). In total, 25 nM of siRNA mix containing siBLM (5′–GAA UCU CAA UGU ACA UAG AUU UU; Dharmacon), siEXO1 (5′–UGC CUU UGC UAA UCC AAU CCC ACG C; Invitrogen) or the siScramble control Dharmacon ON-TARGET plus Non-Targeting Control siRNA 1 Cat. No.: D–001810–01–05) were added to the cells 24 h after plasmid transfection, using RNAiMax regent Lipofectamine™ RNAiMAX Transfection Reagent (Invitrogen, Cat. No.: 13778075).

### Immunofluorescence staining

Cells were treated with 8 Gy radiation and collected after 4 h of incubation at 37 °C. Pre-extraction was done for 10 min on ice with CSK Buffer (10 mM PIPES/KOH [pH 6.8], 100 mM NaCl, 300 mM Sucrose, 1 mM EGTA, 1 mM MgCl_2_, 1 mM DTT, 0.1% Triton X-100) and fixed with paraformaldehyde (PFA) for 15 min at RT. Next, cells were permeabilized for 15 min at RT and incubated with blocking buffer (5% BSA in 1X PBST (Phosphate-Buffered Saline, 0.1% Tween® 20 Detergent; PBST) for 1 h followed by addition of primary antibody against RAD51 (Abnova, Cat. No.: H00005888-B01P; used at a 1:400 dilution), RPA (Anti-RPA32/RPA2 mouse monoclonal antibody (Clone No.: 9H8), Abcam, Cat. No.: ab2175; used at a 1:500 dilution), or 53BP1 (BD Transduction Laboratories, Cat. No.: 612522; used at a 1:800 dilution) O/N at 4 °C. Cells were washed three times with phosphate-buffered saline (PBS) for 5 min each and stained with an Alexa Fluor 594 labeled secondary antibody (Invitrogen Cat. No.: A-11005; used at a 1:500 dilution) for 1 h at RT followed by 3 more PBS washes. Coverslips were mounted using ProLong Diamond Antifade Mountant with DAPI (Thermo Fisher, Cat. No.: S36964) and sealed. Images were acquired on an Olympus FLV3000 inverted laser scanning microscope using acquisition software FV31S-SW. For visualizing GFP–BLM foci, cells were exposed to 8 Gy IR and then fixed with a 2% PFA solution for 15 min at RT. PFA was replaced then with PBS. Slides were mounted and imaged using confocal microscope using ×60 oil immersion objective. For foci analysis, cells with more than 5 foci were considered positive and at least 300 cells were counted over three independent experiments. Image analysis was performed with Analysis FV31s-DT software. Data analysis and graphs were generated with GraphPad Prism version 8.4.3. *p* values were calculated using one-way ANOVA test using SEM.

### Western blots

Cells were lysed in Radioimmunoprecipitation assay buffer (RIPA buffer; Sigma-Aldrich, cat. No.: R0278) and protein quantitation was done using the Pierce BCA Protein Assay Kit (Pierce, Cat. No.: 23250). In total, 50 µg protein samples were run on 4–15% SDS-PAGE gels, protein from the gels were transferred onto NC membrane (Sigma-Aldrich, Cat. No.: GE10600003). NC membranes were blocked using 5% dry milk in TBST (Tris-buffered saline with 0.1% Tween® 20 detergent) and stained with primary antibodies: anti-BLM (Santa Cruz Biotechnology, Cat. No.: SC-365753; used at a 1:2000 dilution; or Bethyl, Cat. No.: A300-100A; used at a 1:1000 dilution), anti-EXO1(Bethyl, Cat. No.: A302-639A; used at a 1:1000 dilution), anti-FLAG (M2) (Sigma Cat. No.: F1804; used at a 1:1000 dilution), anti-Actin (CST, Cat. No.: 12262S; used at a 1:3000 dilution), anti-GFP (Invitrogen, Cat. No.: A-6455; used at a 1:4000 dilution), or Alpha–Tubulin–HRP (Cell Signaling Technology, Cat. No.: CST 11H10; used at a 1:5000 dilution), as indicated, O/N at 4 °C, washed with TBST buffer 3 times for 10 min each and incubated with appropriate secondary antibody (anti-mouse IgG HRP, Invitrogen Cat. No.: A16078; used at a 1:4000 dilution; or anti-rabbit IgG Fc HRP, Cat. No.: A16116; used at a 1:4000 dilution) for 1 h at RT. Membrane was washed three times for 10 min each and developed using Pierce Enhanced Chemiluminescence substrate (Thermo Fisher, Cat. No.: 32109). Blots were imaged on a BioRad Chemidoc imaging system using Image Lab Touch software version 2.4.0.03.

### Clonogenic survival assays

Clonogenic survival assays were performed as described^86^. Briefly 150 Cells were seeded per well in a 12 well plate and treated with the indicated concentrations of Olaparib (Selleckchem, Cat. No: S1060) and MMC (Selleckchem, Cat. No: S8146) and allowed for colony formation for 10 days. Colonies were fixed with methanol:acetic acid (3:1) for 5 min and stained with 0.5% crystal violet in methanol for 15 min. Plates were washed under tap water and dried before colonies were counted. Cells were imaged on a BioRad Chemidoc imaging system using Image Lab Touch software version 2.4.0.03.

### Cell-based reporter assays

Cell-based reporter assays were performed as previously described^49^. Briefly, U2OS cells harboring integrated copies of either DR-GFP or SA-GFP cassette were transfected with siRNA (siBLM + siEXO1) or Scr-siRNA overnight and complemented with vector control (pBI-MCS-Flag), WT-BLM, BLM^Δ1–641^ and BLM^Δ1291–1330^, along with plasmid expressing I-SceI endonuclease. Cells were incubated 72 h post plasmid transfection and % GFP positive cells were analyzed using flow cytometry (BD LSR-II). Repair efficiency was calculated as ${{{{{{\rm{repair}}}}}}\; {{{{{\rm{efficiency}}}}}}}=\left( \% {{{{{{\rm{GFP}}}}}}\; {{{{{\rm{positive}}}}}}}\div{{{{{{\rm{transfection}}}}}}\; {{{{{\rm{efficiency}}}}}}}\right)\times 100$. Experiments were performed in triplicate and *p* values were calculated using one-way ANOVA test using SEM

### PCR-based resection assays

The percentage of DNA resection adjacent to a specific DSB, DSB1 (Chr 1: 89231183) was measured as previously described^50^. Briefly, ER-*Asi*SI U2OS cells were depleted of endogenous BLM and EXO1 using siRNA and then complemented with WT-BLM, BLM^Δ1–641^, or BLM^Δ1291–1330^. Cells were then treated with 300 nM 4-Hydroxytamoxifen (4-OHT; Sigma) for 4 h to allow the nuclear translocation of *AsiSI* and the induction of DSBs. Cells were harvested and genomic DNA extracted using a DNeasy Kit (Qiagen). After that, 1500 ng of genomic DNA was digested, or mock digested, with 2 μl of BsrGI enzyme (New England Biolabs, Cat No. R3575L) in 30 μl total volume at 37 °C overnight. In total, 1 μl of DNA was used as template in 10 μl of qPCR reaction performed using SYBR Green master mix (Applied Biosystem, Cat No. A25776) and 0.5 μM of each primer (DSB1-335-F: 5′-GAA TCG GAT GTA TGC GAC TGA TC; DSB1-335-R: 5′-TTC CAA AGT TAT TCC AAC CCG AT). qPCR data were quantified on a 7900 HT Fast Real-Time PCR system (Applied Biosystems) using SDS2.4 software. For each sample, *∆*${Ct}$was calculated by subtracting the Ct value of the mock-digested sample from the ${Ct}$ value of the digested sample. The percentage of ssDNA was calculated with the following equation: ${{{{{{\rm{ssDNA}}}}}}} \% =1/\left({2}^{\left(\triangle {Ct}-1\right)}+0.5\right)\times 100$. Experiments were performed in quadruplicate and *p* values were calculated using unpaired *t* test.

### Statistical analysis

For the single-molecule BLM binding distribution analysis and error bars represent 95% confidence intervals calculated from bootstrap analysis of the data. The number of single BLM molecules measured (*N*) are specified in each binding distribution histogram. For the single-molecule BLM velocity and processivity data error bars represent standard deviation (SD) and the *N* values are presented in each corresponding figure panel. The statistical parameters (velocity ± SD; processivity ± SD; *N* values; and corresponding figure panels) for all velocity and processivity measurements are summarized in Supplementary Table 1. For the fraction of end looping events shown in Fig. 4f, *N* values are presented in the figure panel and error bars represent SD from three independent experiments. The analysis shown in Supplementary Fig. 2b was done using Pearson’s correlation analysis. Note, that the *N* values for all single-molecule experiments represent the number of single molecules that were analyzed for each given experiment and reflect the cumulative data collected from at least three separate flowcells. *p* values for the single-molecule velocity and processivity assays (Figs. 1e, f, 3b, c, 4c, d, 5e, f and Supplementary 1d, e) were calculated using a two-tailed Student’s *t* test. For the bulk biochemical ATP hydrolysis (Fig. 5d), DNA unwinding (Fig. 6b) and DNA degradation assays (Fig. 6d) the error bars represent SD calculated from three separate reactions. *p* values for the biochemical assays were calculated using a two-tailed Student’s *t* test.

For cell survival assays (Fig. 7a, b), the data points represent the mean ± standard error of the mean (SEM) from three independent experiments. The data for the foci measurement assays (Fig. 7d) represent the mean ± SEM, where *N* equals three independent experiments. For each experiment, cells were scored as positive if they contained greater than five foci per cell and ≥300 cells were analyzed for each different condition. For reporter assays (Fig. 8b, c) the % GFP positive cells were calculated by analyzing at least 10,000 cells from a population. Data points represent the mean ± SEM from three independent experiments and *p* values for cellular assays were calculated using one-way ANOVA test using the SEM.

For PCR-based resection assays (Supplementary Fig. 8b), the data points represent the mean ± SEM from four independent experiments. *p* values were calculated using an unpaired Student’s *t* test using the SEM.

### Reporting summary

Further information on research design is available in the Nature Research Reporting Summary linked to this article.

## Supplementary information

Supplementary Information

Description of Additional Supplementary Information

Reporting summary

## Data Availability

The data supporting the findings of this study are available from the corresponding authors upon reasonable request. Source data are provided with this paper.

## Source data

Source Data
