# Supplementary material for: Bloom helicase mediates formation of large single–stranded DNA loops during DNA end processing
Source: Nat Commun. 2022 Apr 26;13:2248. doi: 10.1038/s41467-022-29937-7 (PMC9042962; doi:10.1038/s41467-022-29937-7)
Supplement: Supplementary file 1 — Supplementary Information [file 41467_2022_29937_MOESM1_ESM.pdf]

Bloom helicase mediates formation of large single–stranded DNA loops during DNA end processing

Chaoyou Xue[1,4†], Sameer J. Salunkhe[2,3†], Nozomi Tomimatsu[4], Ajinkya S. Kawale[2,7], Youngho Kwon[2,3], Sandeep Burma[2,5], Patrick Sung[2,3*] and Eric C. Greene[1*]

[1] Department of Biochemistry & Molecular Biophysics, Columbia University, New York, New York, 10032, USA
[2] Department of Biochemistry and Structural Biology, University of Texas Health Science Center at San Antonio, Texas 78229, USA
[3]The Greehey Children's Cancer Research Institute, University of Texas Health Science Center at San Antonio, San Antonio, TX 78229, USA
[4]Present address: Tianjin Institute of Industrial Biotechnology, Chinese Academy of Sciences, Tianjin 300308, China
[5]Department of Neurosurgery, University of Texas Health Science Center at San Antonio, Texas 78229, USA
[6]Present address: Massachusetts General Hospital Cancer Center, Harvard Medical School, Charlestown, MA 02129, USA
[†]Equal contribution
[*]Correspondence: ecg2108@cumc.columbia.edu and sungp@uthscsa.edu

Includes:

Supplementary Figures 1 through 9

Supplementary Table 1

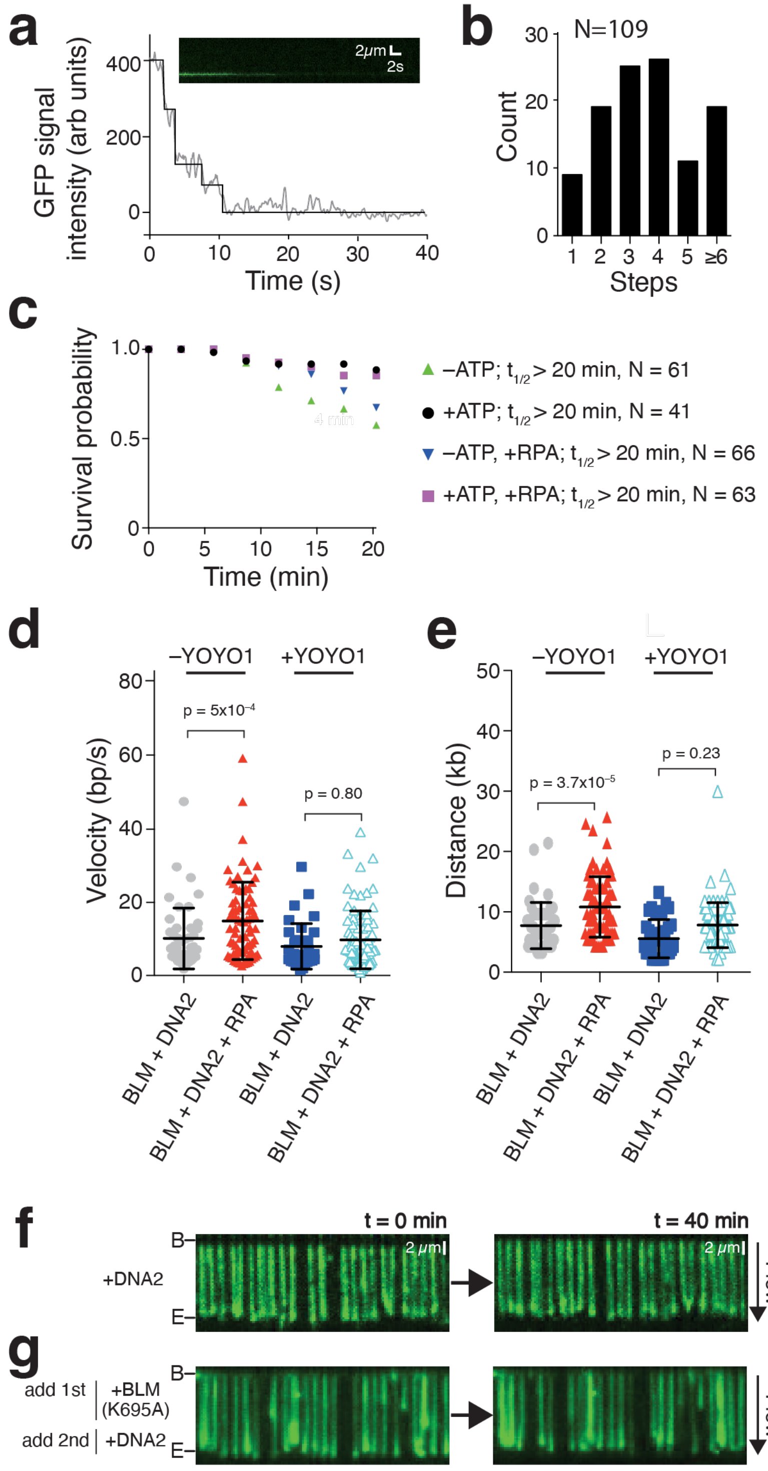

Supplementary Fig. 1. Characterization of end–bound GFP–BLM. (**a**) Graph showing a photo–bleaching step trace for end–bound GFP–BLM. The inset shows the kymograph corresponding to the graphed data. (**b**) Distribution of photo–bleaching steps observed for end–bound GFP–BLM (N=109). (**c**) Survival probability plot showing the lifetimes of end–bound GFP–BLM ± ATP and/or ± RPA, as indicated. (**d**) Velocity distribution for

end–bound GFP–BLM (plus and minus DNA2 or RPA) in the presence or absence of 0.5 nM YOYO–1. The center bar represents the mean of the data and error bars represent SD. P values were calculated using a two–tailed Student's t–test. Also see Table S1. (**e**) Processivity of end–bound GFP–BLM (plus and minus RPA) in the presence or absence of 0.5 nM YOYO–1. The center bar represents the mean of the data and error bars represent SD. P values were calculated using a two–tailed Student's t–test. Also see Table S1. (**f**) Wide–field image of YOYO–1–stained single–tethered dsDNA curtain before (t = 0 min) and after (t = 40 min) the injection of 0.2 nM DNA2 and 2 mM ATP. The dsDNA is shown in green. (**g**) Wide–field image of YOYO–1–stained single–tethered dsDNA curtain before and after the injection of 0.2 nM BLM–K695A, 0.2 nM DNA2 and 2 mM ATP.

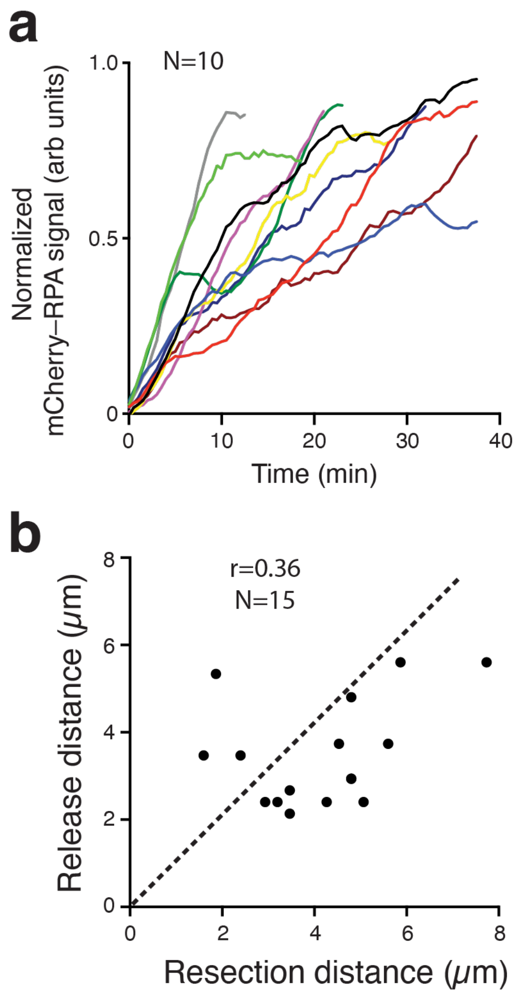

Supplementary Fig. 2. Characterization of ssDNA production and loop release. (**a**) Normalized RPA–mCherry signal accumulation during DNA end resection in reactions with 0.2 nM GFP–BLM, 0.2 nM DNA2 and 2 nM RPA–mCherry. (**b**) Plot showing the relationship between DNA end resection length and the length of RPA–coated ssDNA that was released in single–step release events.

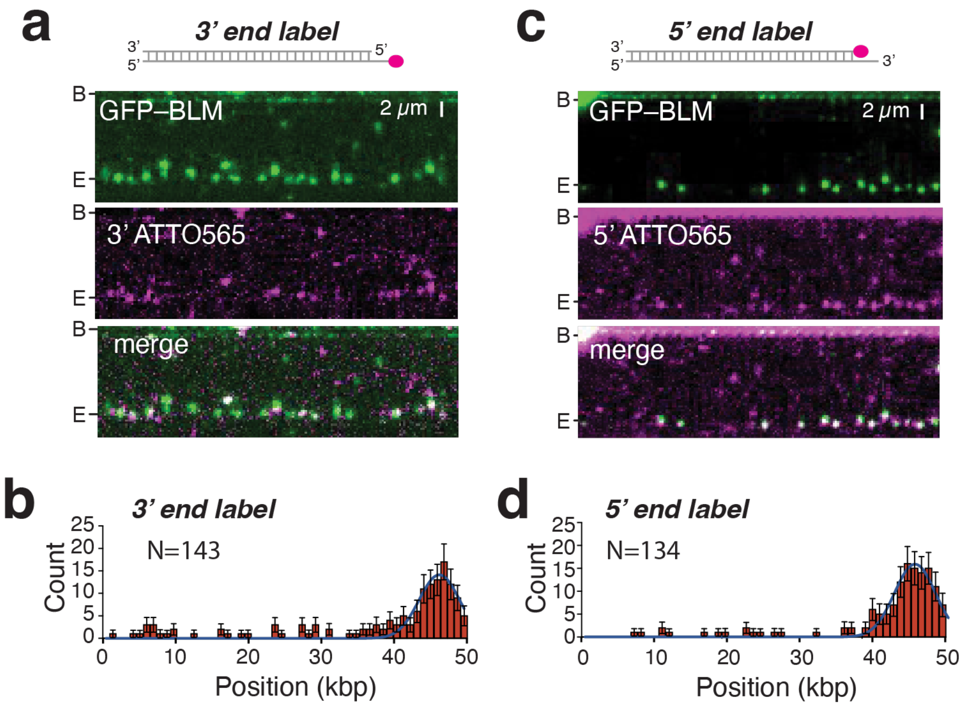

Supplementary Fig. 3. Binding properties of GFP–BLM with 5′ or 3′ ATTO565–labeled DNA. (**a**) Wide–field image of GFP–BLM (green) bound to a 3′ ATTO565–labeled DNA curtain. (**b**) GFP–BLM binding site distribution on the 3′ ATTO565–labeled DNA. The height of each bar represents the cumulative number of GFP–BLM molecules bound within each given bin and error bars represent 95% confidence intervals (CI) calculated from bootstrap analysis. (**c**) Wide–field image of GFP–BLM (green) bound to a 5′ ATTO565–labeled DNA curtain. (**d**) GFP–BLM binding site distribution on the 5′ ATTO565–labeled DNA. The height of each bar represents the cumulative number of GFP–BLM molecules bound within each given bin and error bars represent 95% confidence intervals (CI) calculated from bootstrap analysis.

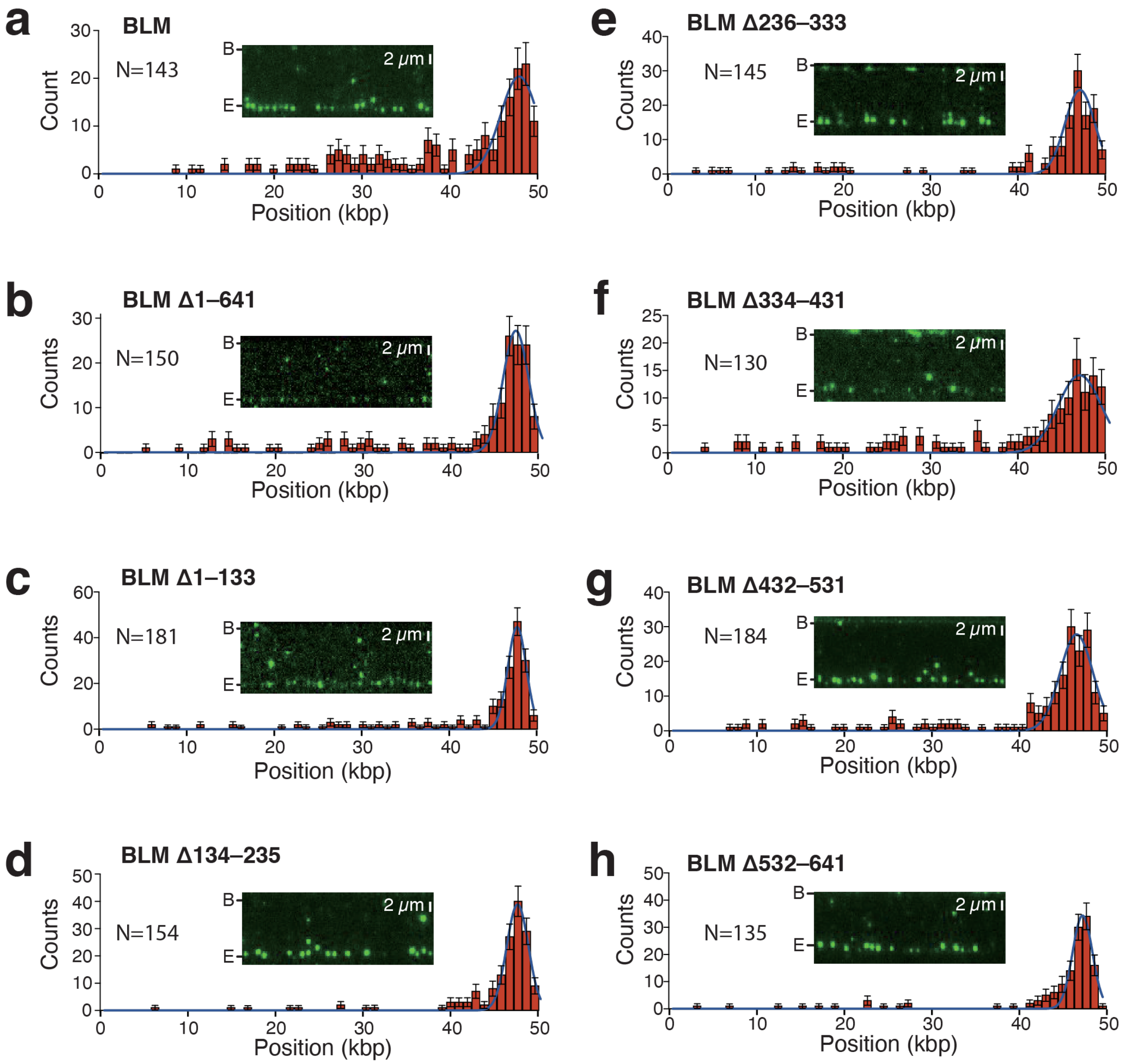

Supplementary Fig. 4. DNA binding properties of BLM NTD truncation mutants. (**a**) Binding site distribution of full–length GFP–BLM bound to a single–tethered dsDNA curtain. Error bars represent 95% CI calculated from bootstrap analysis. The inset shows an example of a wide–field image of GFP–BLM bound to the DNA curtain. Note that this data for full–length GFP–BLM is reproduced from Fig. 1b–c for comparison to the NTD truncation mutants. (**b**–**h**) Binding site distributions of the indicated GFP–BLM truncation mutants bound to a single–tethered dsDNA curtain. The height of each bar represents the cumulative number of GFP–BLM molecules bound within each given bin and error bars represent 95% confidence intervals (CI) calculated from bootstrap analysis. In each case, the inset shows an example of a wide–field image of the indicated GFP–BLM mutant bound to the DNA curtain.

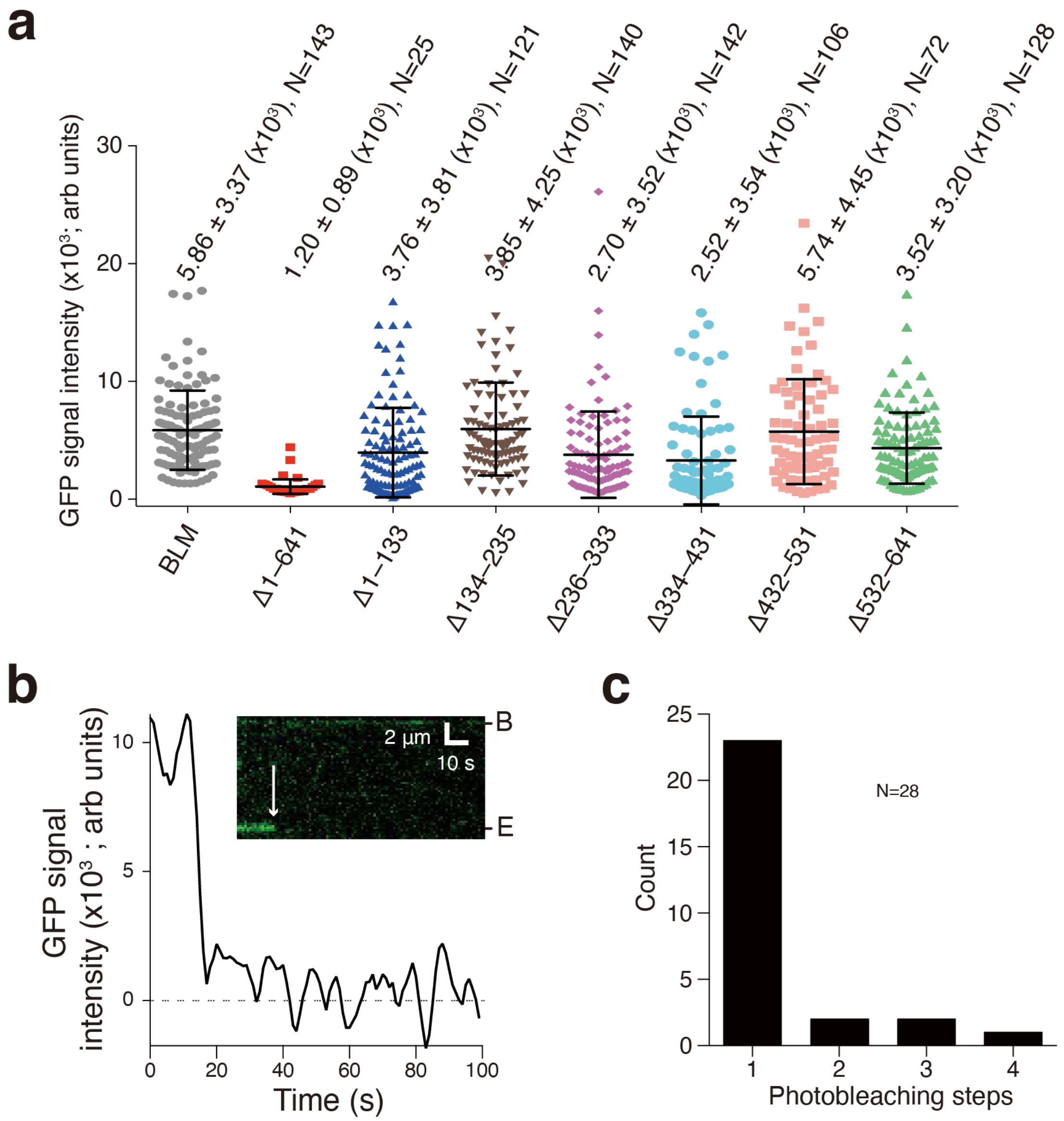

Supplementary Fig. 5. Characterization of the end–bound GFP–BLM NTD truncation mutants. (**a**) Comparison of the integrated GFP signal intensity (arbitrary units) for GFP–tagged versions of each indicated BLM construct bound to the DNA ends in a DNA curtain. The center bar represents the mean of the data and error bars represent SD. (**b**) Graph showing a photo–bleaching step trace for end–bound GFP–BLM$^{\Delta 1-641}$. The inset shows the kymograph corresponding to the graphed data. (**c**) Distribution of photo–bleaching steps observed for end–bound GFP–BLM$^{\Delta 1-641}$ (N = 28).

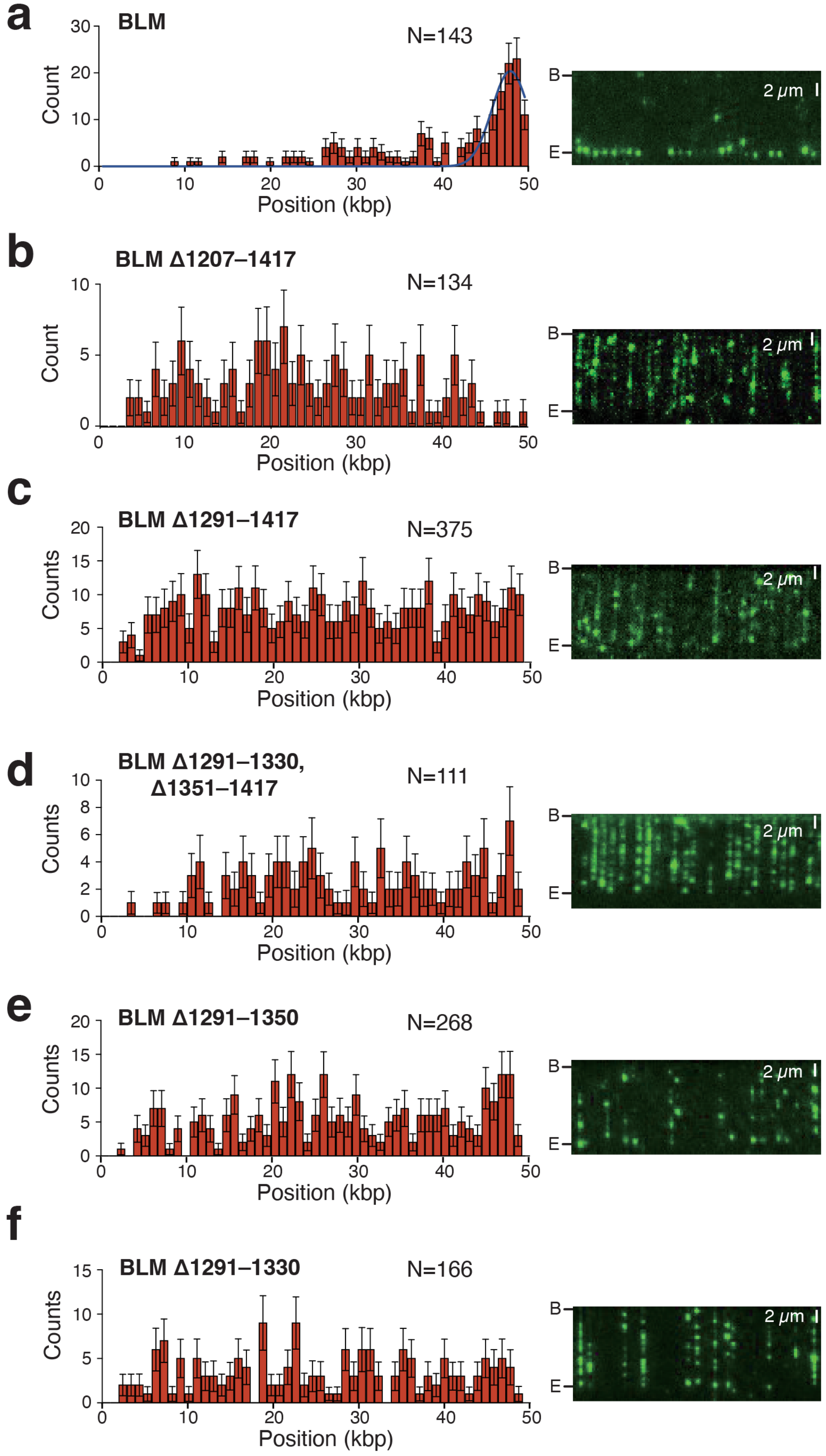

a
BLM
N=143
Count
Position (kbp)
2 μm
B
E
b
BLM Δ1207–1417
N=134
Count
Position (kbp)
c
BLM Δ1291–1417
N=375
Counts
Position (kbp)
d
BLM Δ1291–1330,
Δ1351–1417
N=111
Counts
Position (kbp)
e
BLM Δ1291–1350
N=268
Counts
Position (kbp)
f
BLM Δ1291–1330
N=166
Counts
Position (kbp)

Supplementary Fig. 6. The CTD of BLM regulates DNA end recognition. (**a**) Binding site distribution of full–length GFP–BLM bound to a single–tethered dsDNA curtain. Error bars represent 95% CI calculated from bootstrap analysis. The right panel shows an example of a wide–field image of GFP–BLM bound to the DNA curtain. Note that this data for full–length GFP–BLM is reproduced from Fig. 1B for comparison to the CTD truncation mutants. (**b**–**f**) Binding site distributions of the indicated GFP–BLM truncation mutants bound to a single–tethered dsDNA curtain. The height of each bar represents the cumulative number of GFP–BLM molecules bound within each given bin and error bars represent 95% confidence intervals (CI) calculated from bootstrap analysis. In each case, the right panel shows an example of a wide–field image of the indicated GFP–BLM mutant bound to the DNA curtain.

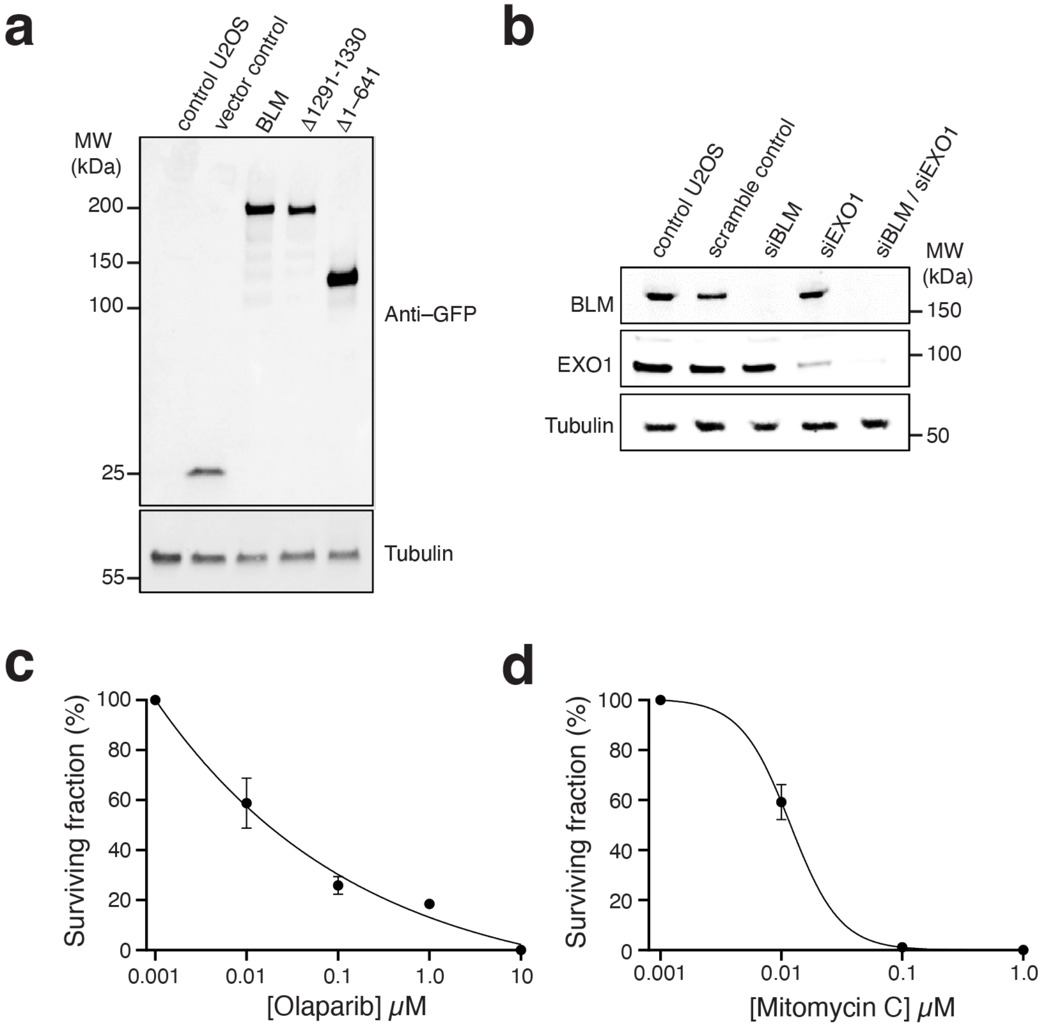

Supplementary Fig. 7. Controls for in vivo analysis of BLM truncation mutants. (a) Western blots of extracts from U2OS cells reconstituted with the indicated genotypes immuno–stained with anti–GFP and anti–Tubulin HRP antisera, as indicated. **(b)** Immunoblot demonstrating siRNA knockdown of either BLM, EXO1 or both. Graphs showing the % survival for U2OS cells without BLM or EXO1 depletion following treatment with **(c)** Olaparib or **(d)** Mitomycin C for IC50 determination as assessed by clonogenic survival assays in U2OS cells expressing full–length GFP–tagged BLM. Data points represent the mean ± SEM for N = 3 separate assays.

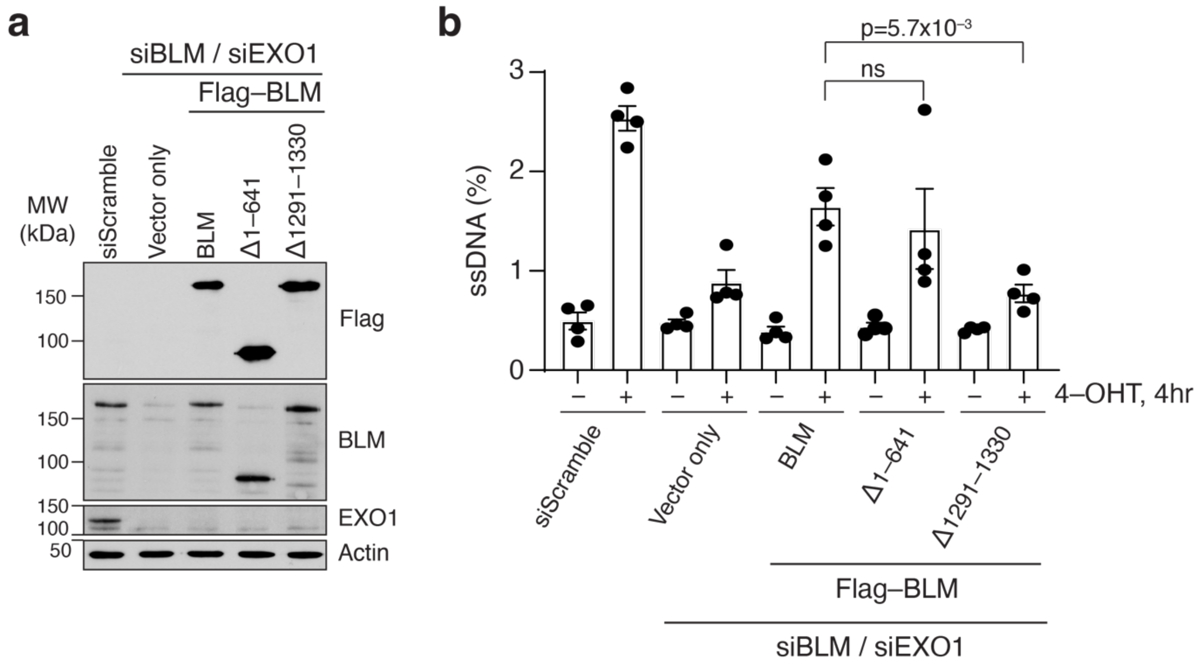

Supplementary Fig. 8. PCR–based detection of end resection in cells expressing BLM$^{\Delta 1–641}$ or BLM$^{\Delta 1291–1330}$. (**a**) Western blot showing siRNA–mediated depletion of endogenous EXO1 and BLM, and ectopic expression of Flag–tagged full–length BLM, or the indicated BLM deletion constructs, in ER–*Asi*SI U2OS cells, as indicated. (**b**) Genomic DNA extracted from 4–OHT treated cells were mock–digested or digested with BsrGI, and DNA end resection was quantified by qPCR. The plot shows ssDNA percentages adjacent to DSBs (335 nt) in cells expressing the indicated BLM proteins. Data points represent the mean ± SEM for N = 4 separate assays. P values were calculated using a two–tailed Student's t–test.

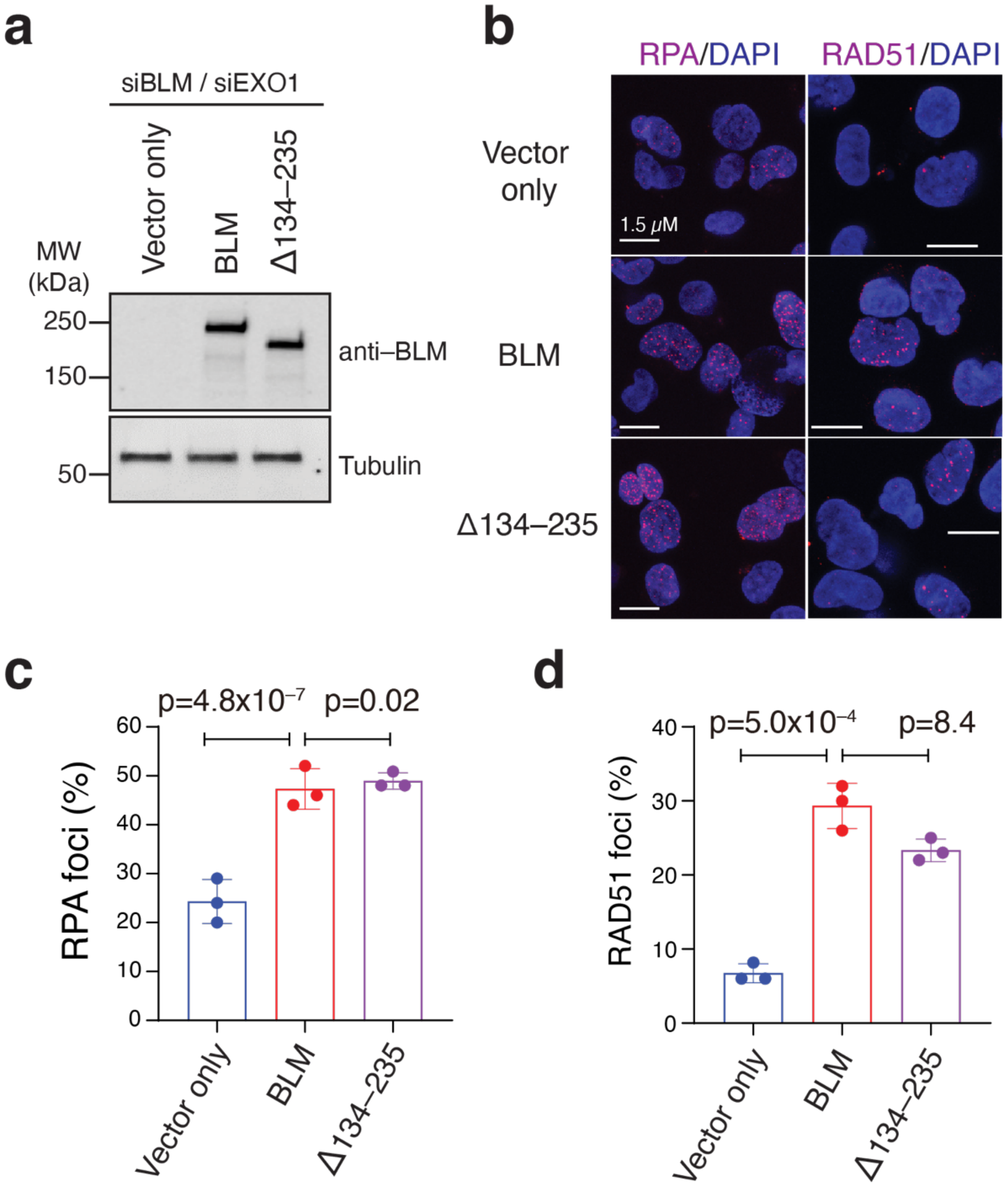

Supplementary Fig. 9. DNA repair foci formation in cells expressing BLM$^{\Delta 134–235}$. (**a**) Western blots of extracts from U2OS cells depleted for BLM and EXO1 were reconstituted with the indicated BLM constructs and stained with anti–BLM or anti–Tubulin antisera, as indicated. (**b**) Images of cells stained with DAPI and immunostained with antisera against RPA or RAD51, as indicated, 4 hours after exposure to 8 Gy ionizing radiation. (**c**) Quantitation of RPA foci and (**d**) Quantitation of RAD51 foci. In (c) and (d), The data points represent the fraction (%) or cells (mean ± SEM, N = three independent experiments) with greater than five foci per cell (≥300 cells were analyzed per condition); P values were calculated using one–way ANOVA test.

Table S1. Summary of BLM translocation parameters.

	Reaction Conditions	Velocity (bp/s)	Processivity (kb)	N	Fig
**End–bound**	GFP–BLM + DNA2	10 ± 8	7.1 ± 2.7	48	1E–F
	GFP–BLM + DNA2 + RPA	15 ± 10	10.8 + 5.0	76	1E–F
	GFP–BLM + RPA–mCherry	40 ± 23	10.1 ± 5.5	64	3B–C
	GFP–BLM + RPA–GFP	35 ± 17	12.1 ± 6.1	107	4C–D
	GFP–BLM$^{\Delta 1–641}$ + RPA–GFP	54 ± 18	28.0 ± 12.0	101	4C–D
	GFP–BLM$^{\Delta 1–133}$ + RPA–GFP	46 ± 24	16.5 ± 10.5	119	4C–D
	GFP–BLM$^{\Delta 134–235}$ + RPA–GFP	43 ± 26	14.1 ± 8.7	163	4C–D
	GFP–BLM$^{\Delta 236–333}$ + RPA–GFP	41 ± 22	14.5 ± 9.2	154	4C–D
	GFP–BLM$^{\Delta 334–431}$ + RPA–GFP	47 ± 22	14.9 ± 10.4	138	4C–D
	GFP–BLM$^{\Delta 432–531}$ + RPA–GFP	40 ± 20	15.4 ± 9.4	112	4C–D
	GFP–BLM$^{\Delta 532–641}$ + RPA–GFP	37 ± 23	12.3 ± 10.5	137	4C–D
	GFP–BLM + DNA2 + YOYO–1	8 ± 6	5.7 ± 3.2	35	S1D–E
	GFP–BLM + DNA2 + RPA + YOYO–1	10 ± 8	7.8 ± 3.7	82	S1D–E
**Internal–bound**	‡GFP–BLM + RPA	101 ± 35	8.1 ± 4.1	99	5E–F
	GFP–BLM$^{\Delta 1207–1417}$ + RPA	36 ± 20	8.1 ± 4.4	95	5E–F
	GFP–BLM$^{\Delta 1291–1417}$ + RPA	90 ± 33	9.2 ± 4.1	115	5E–F
	GFP–BLM$^{\Delta 1291–1330,\ \Delta 1351–1417}$ + RPA	89 ± 29	9.8 ± 4.7	99	5E–F
	GFP–BLM$^{\Delta 1291–1350}$ + RPA	85 ± 42	10.2 ± 4.9	77	5E–F
	GFP–BLM$^{\Delta 1291–1330}$ + RPA	82 ± 33	10.6 ± 4.4	76	5E–F

‡Note, the data parameters for GFP–BLM + RPA bound to internal DNA sites are from ref. 17. and shown here for comparison
