# Supplementary material for: Bloom helicase mediates formation of large single–stranded DNA loops during DNA end processing
Source: Nat Commun. 2022 Apr 26;13:2248. doi: 10.1038/s41467-022-29937-7 (PMC9042962; doi:10.1038/s41467-022-29937-7)
Supplement: Supplementary file 2 — Description of Additional Supplementary Information [file 41467_2022_29937_MOESM2_ESM.pdf]

Description of Additional Supplementary Information

1. Supplementary Information (PDF): Includes all nine supplemental figures and a supplemental Table.
2. Supplementary Data 1 (PDF): Includes full scans of all gels and western blots from this study.
3. Source Data (xlsx): Includes all raw data values from this study organized by figure and figure panel.
