# Supplementary material for: Bloom helicase mediates formation of large single–stranded DNA loops during DNA end processing
Source: Nat Commun. 2022 Apr 26;13:2248. doi: 10.1038/s41467-022-29937-7 (PMC9042962; doi:10.1038/s41467-022-29937-7)
Supplement: Supplementary file 3 — Reporting summary [file 41467_2022_29937_MOESM3_ESM.pdf]

nature portfolio

Corresponding author(s): Eric C Greene

Last updated by author(s): Feb 23, 2022

Reporting Summary

Nature Portfolio wishes to improve the reproducibility of the work that we publish. This form provides structure for consistency and transparency in reporting. For further information on Nature Portfolio policies, see our Editorial Policies and the Editorial Policy Checklist.

Statistics

For all statistical analyses, confirm that the following items are present in the figure legend, table legend, main text, or Methods section.

n/a	Confirmed	
☐	☒	The exact sample size (n) for each experimental group/condition, given as a discrete number and unit of measurement
☐	☒	A statement on whether measurements were taken from distinct samples or whether the same sample was measured repeatedly
☐	☒	The statistical test(s) used AND whether they are one- or two-sided<br>*Only common tests should be described solely by name; describe more complex techniques in the Methods section.*
☐	☒	A description of all covariates tested
☐	☒	A description of any assumptions or corrections, such as tests of normality and adjustment for multiple comparisons
☐	☒	A full description of the statistical parameters including central tendency (e.g. means) or other basic estimates (e.g. regression coefficient) AND variation (e.g. standard deviation) or associated estimates of uncertainty (e.g. confidence intervals)
☐	☒	For null hypothesis testing, the test statistic (e.g. F, t, r) with confidence intervals, effect sizes, degrees of freedom and P value noted<br>*Give P values as exact values whenever suitable.*
☒	☐	For Bayesian analysis, information on the choice of priors and Markov chain Monte Carlo settings
☒	☐	For hierarchical and complex designs, identification of the appropriate level for tests and full reporting of outcomes
☒	☐	Estimates of effect sizes (e.g. Cohen's d, Pearson's r), indicating how they were calculated

Our web collection on statistics for biologists contains articles on many of the points above.

Software and code

Policy information about availability of computer code

Data collection	Single molecule data were collected using NIS-Elements Version 5.11 (Nikon). Immunofluorescence images were acquired on Olympus fluoview FV 3000 using acquisition FV31S-SW software. Western blot and clonogenic survival assay images were acquired on Biorad chemidoc imaging system using Image lab touch software version2.4.0.03. qPCR data were quantified on 7900 HT Fast Real-Time PCR system (Applied Biosystems) using SDS2.4 software.
Data analysis	Single molecule image analysis was performed using Open-source image processing software ImageJ. Immunofluorescence imaging data was analyzed using Analysis FV31s-DT software. Data analysis and graphs were plotted using GraphPad Prism version 8.4.3 (686).

For manuscripts utilizing custom algorithms or software that are central to the research but not yet described in published literature, software must be made available to editors and reviewers. We strongly encourage code deposition in a community repository (e.g. GitHub). See the Nature Portfolio guidelines for submitting code & software for further information.

Data

Policy information about availability of data

All manuscripts must include a data availability statement. This statement should provide the following information, where applicable:

- Accession codes, unique identifiers, or web links for publicly available datasets
- A description of any restrictions on data availability
- For clinical datasets or third party data, please ensure that the statement adheres to our policy

The data that support the findings of this study are available from the corresponding authors upon reasonable request.

Field-specific reporting

Please select the one below that is the best fit for your research. If you are not sure, read the appropriate sections before making your selection.

☒ Life sciences ☐ Behavioural & social sciences ☐ Ecological, evolutionary & environmental sciences

For a reference copy of the document with all sections, see nature.com/documents/nr-reporting-summary-flat.pdf

Life sciences study design

All studies must disclose on these points even when the disclosure is negative.

Sample size	Sample sizes were not preselected. All single molecule measurements reflect the cumulative results from tens to hundreds of individual molecules, which is consistent with expectations within the field (all exact n values and associated statistical parameters are reported in the manuscript). For cell survival assays, the data points represent the mean ± standard error of the mean (SEM) from three independent experiments. The data for the foci measurement assays ¬represent the mean ± SEM, where N equals three independent experiments. For each experiment, cells were scored as positive if they contained greater than five foci per cell and ≥300 cells were analyzed for each different condition. P values for cellular assays were calculated using one–way ANOVA test using the SEM.
Data exclusions	Data were not excluded.
Replication	All reported data represent a minimum of three experimental replicates to ensure reproducibility.
Randomization	Randomization was not part of the study design.
Blinding	Blinding was not part of the study design. This research involved molecular analysis of specific protein mutants and the researchers needed to make and characterize each known mutant independently.

Reporting for specific materials, systems and methods

We require information from authors about some types of materials, experimental systems and methods used in many studies. Here, indicate whether each material, system or method listed is relevant to your study. If you are not sure if a list item applies to your research, read the appropriate section before selecting a response.

Materials & experimental systems

n/a	Involved in the study	
☐	☒	Antibodies
☐	☒	Eukaryotic cell lines
☒	☐	Palaeontology and archaeology
☒	☐	Animals and other organisms
☒	☐	Human research participants
☒	☐	Clinical data
☒	☐	Dual use research of concern

Methods

n/a	Involved in the study	
☒	☐	ChIP-seq
☒	☐	Flow cytometry
☒	☐	MRI-based neuroimaging

Antibodies

Antibodies used	(1) Anti–BLM, Santa Cruz Biotechnology, Cat. No.: SC–365753; (2) Anti–EXO1, Bethyl, Cat. No.: A302–639A; (3) Alpha–Tubulin–HRP, Cell Signaling Technology, Cat. No.: CST 11H10; (4) Anti-RAD51, Abnova, Cat. No.: H00005888–B01P; (5) Anti–RPA32/RPA2 mouse monoclonal antibody, Abcam, Cat. No.: ab2175; (6) Anti-53BP1, BD Transduction Laboratories, Cat. No.: 612522, (7) Anti-BLM, Bethyl, Cat. No.: A300–100A, (8) Anti-Flag (M2), Sigma, Cat. No.: F1804, (9) Anti-Actin, CST, Cat. No.: 12262S.
Validation	All antibodies used are commercially available and validated by the manufacturer.

Eukaryotic cell lines

Policy information about cell lines

Cell line source(s)	U2OS cells were purchased from the American Type Culture Collection (ATCC). Reporter assay cell lines (DR–GFP or SA–GFP) were obtained from Prof. Jeremy Stark (Department of Cancer Genetics and Epigenetics, City of Hope Comprehensive Cancer Center).
Authentication	U2OS cells were acquired directly from the American Type Culture Collection (ATCC) and were not authenticated. The U2OS reporter cell lines were provided by Dr. Jeremy Stark (City of Hope), which were generated using U2OS cells acquired directly from the American Type Culture Collection (ATCC).

Mycoplasma contamination	All the cell lines were tested for mycoplasma contamination using MycoAlertTM Mycoplasma Detection Kit (Catalog #: LT07-318) and we confirm that all cell lines tested negative for mycoplasma contamination.
Commonly misidentified lines (See ICLAC register)	Not applicable.
