# Supplementary material for: Bloom helicase mediates formation of large single–stranded DNA loops during DNA end processing
Source: Nat Commun. 2022 Apr 26;13:2248. doi: 10.1038/s41467-022-29937-7 (PMC9042962; doi:10.1038/s41467-022-29937-7)

**Full scan of Figure panel 6a; boxed in area corresponds to image shown in the main figure.**

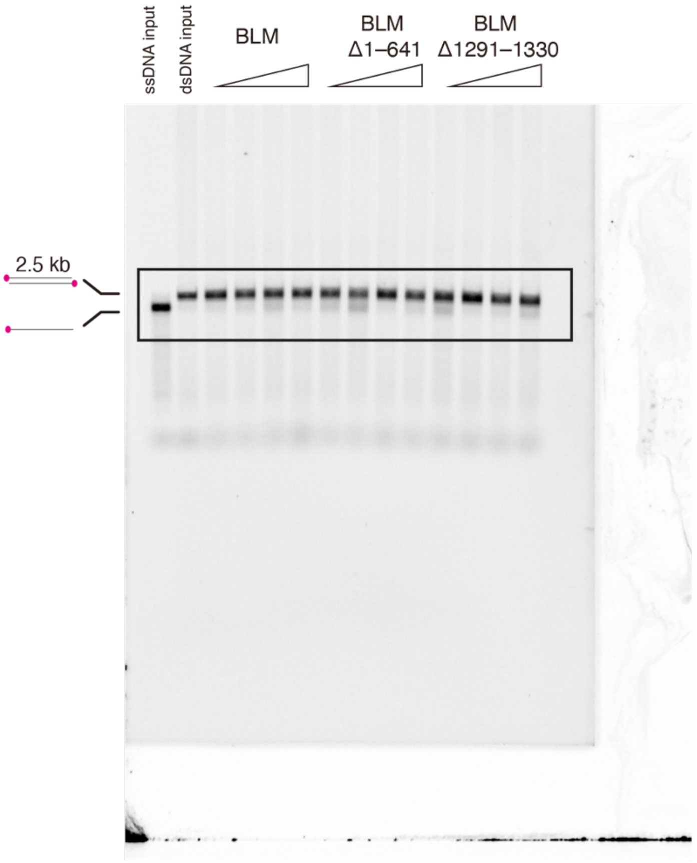

**Full scan of Figure panel 8a; boxed in areas correspond to images shown in the main figure.**

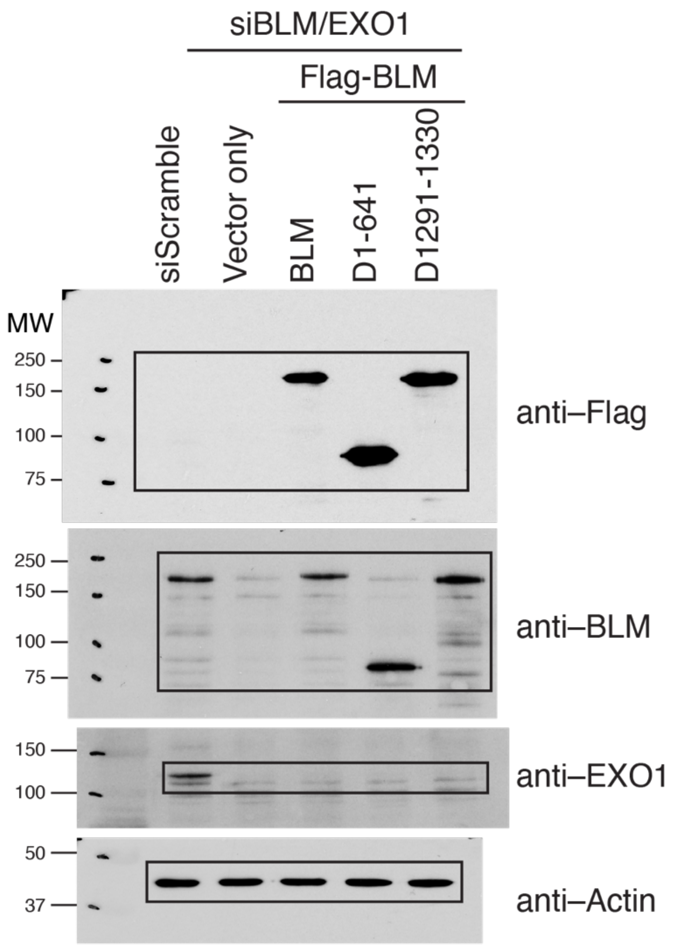

**Full scan of Figure panel S7a; boxed in areas correspond to images shown in the main figure.**

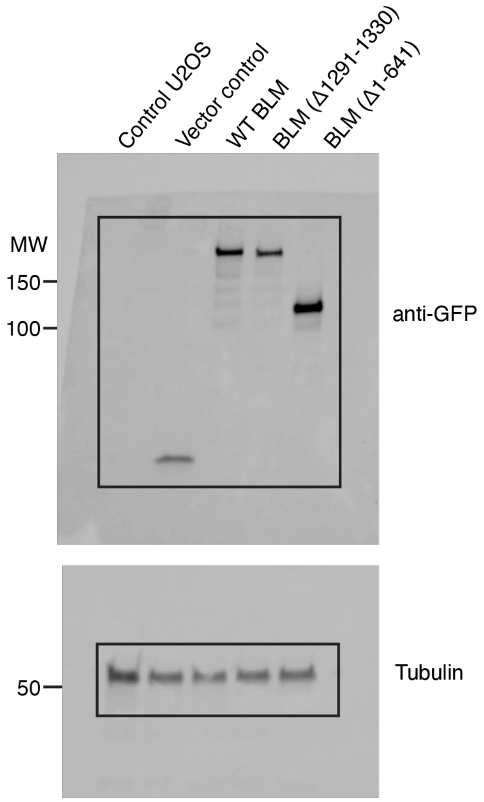

**Full scan of Figure panel S7b; boxed in areas correspond to images shown in the main figure.**

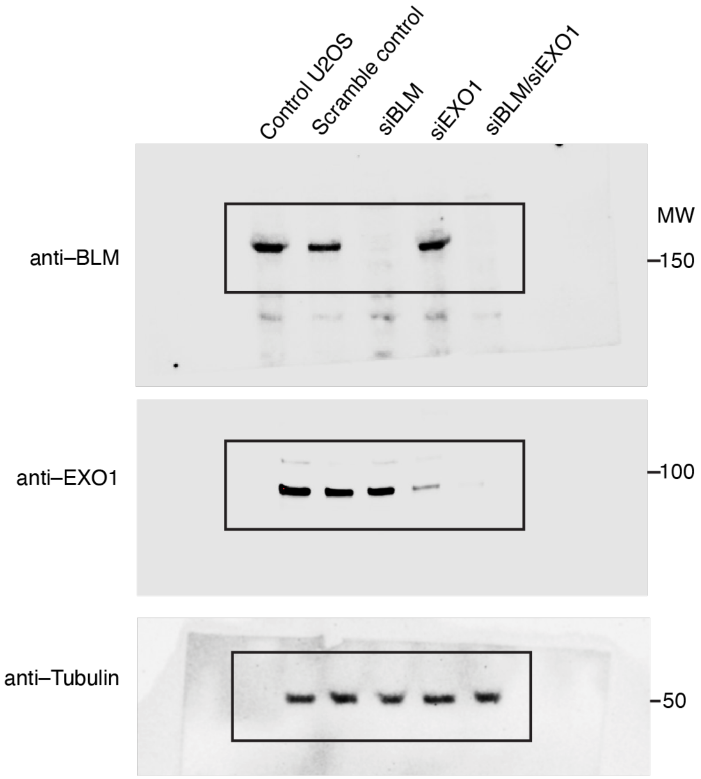

**Full scan of Figure panel S8a; boxed in areas correspond to images shown in the main figure.**

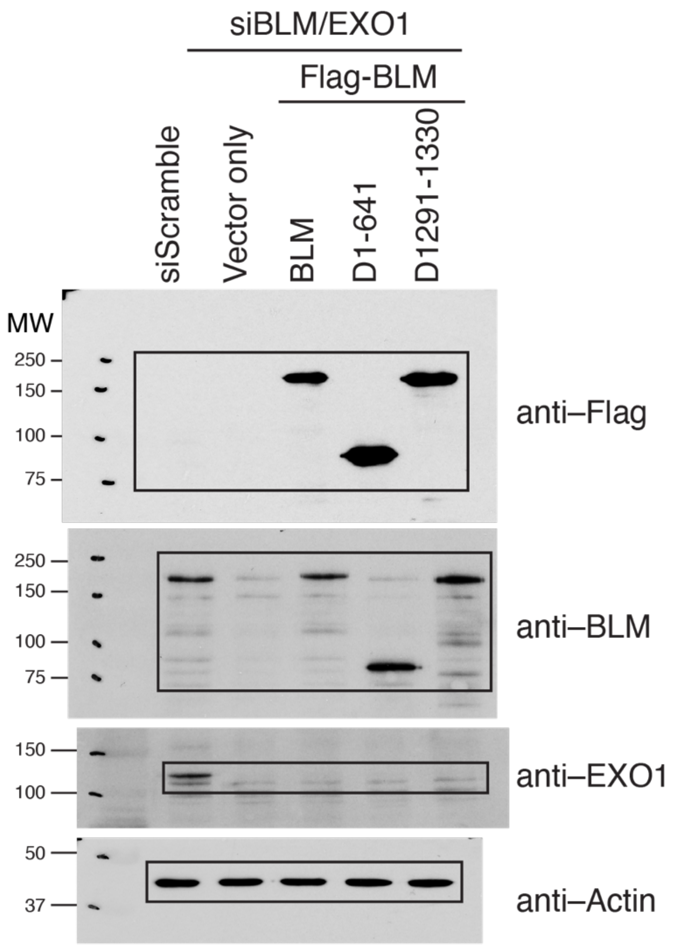

**Full scan of Figure panel S9a; boxed in areas correspond to images shown in the main figure.**

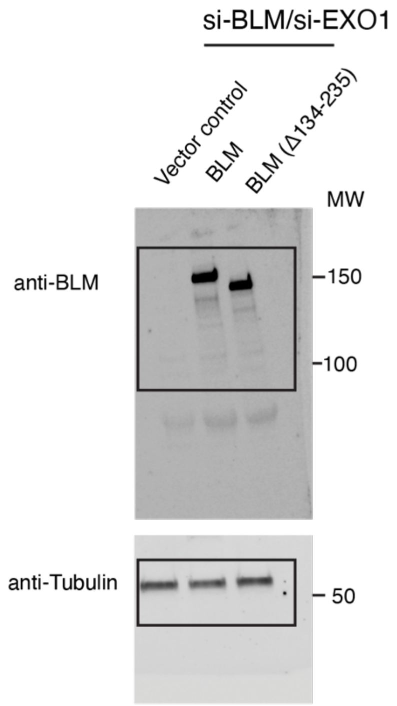

Supplement: Supplementary file 4 — Source Data [file 41467_2022_29937_MOESM4_ESM.zip › Source data/302456_2_data_set_6360029_r8hwkl.pdf]
